# Pharmacological Evaluation of Methanolic Fruit Extract of *Lannea coromandelica*: Antioxidant, Cytotoxic, Antidiabetic, Antibacterial, Anti‐Inflammatory, and Sedative Activities

**DOI:** 10.1002/fsn3.71858

**Published:** 2026-05-05

**Authors:** Priota Islam Meem, Zobayed Islam, Nazmul Hasan Eshaque, Md. Jahirul Islam Mamun, Khurram Murad Mojidee, Mohammad Badrudduja, Amit Kumar Dam, S. M. Moazzem Hossen

**Affiliations:** ^1^ Department of Pharmacy, Faculty of Biological Sciences University of Chittagong Chittagong Bangladesh; ^2^ Department of Applied Chemistry and Chemical Engineering University of Chittagong Chittagong Bangladesh; ^3^ Department of Chemistry University of Dhaka Dhaka Bangladesh

**Keywords:** antibacterial, antioxidant, anti‐inflammatory, cytotoxic, *Lannea coromandelica*, molecular docking, sedative

## Abstract

*Lannea coromandelica* (Anacardiaceae) has been traditionally used in Ayurvedic medicine to treat various ailments. However, its pharmacological potential remains largely unverified. This study investigates the methanolic fruit extract of *L. coromandelica* (ME) through in vitro, in vivo, in silico, and GC–MS analyses to validate its traditional uses. The extract was assessed for its total phenolic and flavonoid content, and phytoconstituents were identified using GC–MS profiling. Antioxidant activity was measured by DPPH and ABTS assays, while cytotoxicity was evaluated using brine shrimp lethality and HeLa cell viability assays. Antidiabetic activity was assessed by α‐amylase inhibition, antibacterial properties were tested by agar well diffusion, and anti‐inflammatory and sedative effects were evaluated in Swiss albino mice using carrageenan‐induced paw edema and behavioral tests. Molecular docking studies targeted SUR‐1, DNA gyrase B, caspase‐3, COX‐2, and the GABA‐A receptor. ME exhibited significant phenolic (35 ± 1.03 mg/g) and flavonoid (11.57 ± 0.37 mg/g) content, with GC–MS analysis identifying phenolics, terpenoids, and fatty acid derivatives. The extract demonstrated strong antioxidant (IC_50_: 31.49 and 48.94 μg/mL), cytotoxic (LC_50_: 36.22 μg/mL; < 5% HeLa survival), and α‐amylase inhibitory (IC_50_: 93.09 μg/mL) activities. It also showed broad‐spectrum antibacterial effects, excellent anti‐inflammatory activity, and significant sedative effects (*p* < 0.001). Molecular docking revealed strong ligand‐target interactions. These results support the pharmacological potential of ME, validating its traditional uses and suggesting its promise for developing therapies targeting inflammation, infection, metabolic, and neurological diseases. Further preclinical and clinical studies are recommended.

## Graphical abstract

Phytochemical investigation of *Lannea coromandelica* through GC–MS and in silico analysis revealed diverse bioactive compounds with promising pharmacological potential. Integrated in vitro and in vivo assays demonstrated significant antioxidant, antidiabetic, antibacterial, anti‐inflammatory, and sedative activities, supporting its therapeutic relevance.

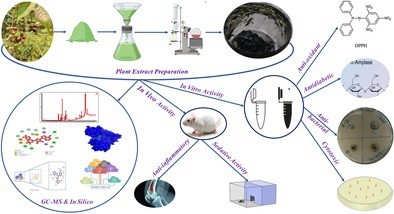

## 1 Introduction

The growing emphasis on sustainability and green chemistry has propelled natural, plant‐derived materials to the forefront across multiple industries, including pharmaceuticals, cosmetics, food, and biodegradable packaging, due to their renewability, biocompatibility, low toxicity, and minimal environmental footprint. Plant‐based resources offer not only eco‐friendly alternatives to synthetic compounds but also a rich reservoir of bioactive molecules with multifunctional properties, aligning with global demands for safer, circular, and ethically sourced solutions (Khalid et al. 2025). Natural products are gaining attention due to their bioactive components and low toxicity (Hasan et al. 2025). Plants with medicinal properties have been used as conventional healing agents since ancient times, even before the discovery of synthetic drugs. Although modern medicine has developed, people continue to use these plants due to their effectiveness, affordability, and minimal side effects (Chirumamilla and Taduri 2023). Medicinal plants contain bioactive compounds like phenols, flavonoids, alkaloids, terpenoids, glycosides, and essential oils, offering diverse therapeutic effects and widespread use in preventing various diseases (Lokapur et al. 2020; Aguerd et al. 2025; Raza et al. 2025). According to the World Health Organization (WHO), nearly 80% of the global population still applies herbal remedies as primary medical care (Chirumamilla and Taduri 2023).

*Lannea coromandelica*, a large tropical tree of the Anacardiaceae family, is widely distributed across Bangladesh, India, Myanmar, Nepal, and Pakistan. Traditionally used in Ayurvedic and Siddha medicine, its bark, leaves, fruits, gum, and sap are rich in bioactive compounds, including proteins, carbohydrates, terpenoids, polyphenols, and flavonoids. These phytochemicals contribute to its therapeutic potential, and tribal communities in Bangladesh, including the Garo, Pahan, and Teli, utilize different parts of the plant to manage a variety of health conditions (Alam et al. 2017; Gunjal et al. 2021). Traditionally, *L. coromandelica* fruit sap treats respiratory issues, leaf juice manages ulcers and pain, and bark decoctions address diarrhea, toothache, and indigestion. Recent studies confirm these uses, showing its extracts have antioxidant, antidiabetic, cytotoxic, antidiarrheal, anti‐inflammatory, and antibacterial activities (Alam et al. 2017).

Oxidation is essential for energy production, but it generates reactive oxygen species (ROS) as byproducts. At low levels, ROS act as signaling molecules; however, excess ROS causes oxidative stress, damaging DNA, lipids, and proteins—contributing to diseases like cancer, diabetes, and cardiovascular disorders. The body counters ROS with endogenous antioxidants (e.g., catalase and glutathione reductase), but these may be insufficient under stress, necessitating exogenous sources. With synthetic antioxidants raising safety concerns due to potential carcinogenicity, natural antioxidants from dietary sources are increasingly sought as safer alternatives (Hossen et al. 2022). Natural antioxidants such as vitamins C and E, glutathione, carotenoids, and polyphenols can help counteract oxidative damage and related diseases. Many phytochemicals exhibit multiple therapeutic effects, making them promising candidates for multifunctional, plant‐derived drugs (Dehghan et al. 2016). Diabetes mellitus is a chronic metabolic disorder characterized by hyperglycemia, resulting from inadequate insulin secretion or insulin resistance, with contributing factors including lifestyle, obesity, and genetic predisposition. It is a growing global health concern, particularly in low‐ and middle‐income countries, due to its high impact on morbidity and mortality. The World Health Organization (WHO) projects that by 2045, the global prevalence of diabetes will rise to approximately 693 million adults, underscoring its escalating burden as a major public health crisis (Cho et al. 2018; Aguerd et al. 2025). While drugs such as acarbose, miglitol, and voglibose are commonly used, they often cause gastrointestinal side effects, including diarrhea, bloating, and gas, highlighting the need for safer, more effective plant‐based therapies for diabetes management (Dedvisitsakul and Watla‐iad 2022; Yikna and Yehualashet 2021). Cancer results from genetic and epigenetic alterations that lead to uncontrolled cell proliferation, with nearly 20 million new cases reported annually, particularly affecting low‐ and middle‐income countries. ROS drives tumor progression by inducing DNA mutations, activating oncogenic pathways (e.g., Ras/ERK, PI3K/Akt), promoting angiogenesis, and aiding immune evasion, although high ROS levels can trigger cancer cell death. Despite advances in diagnosis and treatment, cancer remains a leading cause of mortality worldwide. Notably, around 3000 plant species exhibit anticancer activity, highlighting the potential of phytochemicals to develop new, resistance‐breaking therapies (Canga et al. 2022). Bacterial infections, driven by factors like poor sanitation and crowded conditions, pose a global health concern. With rising antibiotic resistance, natural antibacterial agents are increasingly important, as they can inhibit bacterial growth and metabolism. Inflammation is a natural protective response, but chronic inflammation can damage tissues and contribute to diseases like arthritis, heart disease, diabetes, and neurological disorders. While drugs exist, rural populations often rely on medicinal plants due to limited access and the side effects of conventional anti‐inflammatory therapies (Nunes et al. 2020). Studies confirm the anti‐inflammatory effects of plants, highlighting their experimental and cultural significance (Khalid et al. 2025). Insomnia, or sleeplessness, is a sleep disorder marked by difficulties in beginning or sustaining sleep for the necessary period. Insomnia affects a large proportion of the world's population, with more than 30% experiencing sleep disorders (Mai and Buysse 2008). Diazepam is a commonly used sedative drug that is potent but induces severe adverse effects like drowsiness, mental dullness, and even respiratory issues at high doses. Due to such drawbacks, healthier and more powerful drugs, namely, those derived from medicinal herbs, have been in the research hot seat for the treatment of insomnia (Sultana et al. 2018).

Despite its documented ethnomedicinal uses, the fruit of *L. coromandelica* remains significantly underexplored, with only sparse reports on limited bioactivities (e.g., antibacterial and nephroprotective effects) and a notable absence of comprehensive phytochemical characterization. Critically, prior studies lacked GC–MS‐based metabolite profiling, detailed phytochemical screening, and systematic evaluation of key pharmacological properties, including antioxidant, antidiabetic, anti‐inflammatory, sedative, cytotoxic, and antibacterial activities. This study bridges these knowledge gaps through an integrated approach combining in vitro, in vivo, and in silico methodologies to rigorously assess the methanolic fruit extract of *L. coromandelica* (ME). Specifically, we target five therapeutically relevant proteins—sulfonylurea receptor‐1 (SUR‐1), DNA gyrase B, Caspase‐3, COX‐2, and the GABA_A_ receptor to elucidate ME's molecular mechanisms and multitarget potential. By linking phytochemical constituents (via GC–MS) to biological activities and computational docking, this work not only validates the traditional use of *L. coromandelica* fruit but also highlights its promise as a source of novel lead compounds for future drug development.

## 2 Materials and Methods

### 2.1 Chemicals, Reagents, and Drugs

Square Pharmaceuticals Limited is a Bangladeshi pharmaceutical company that supplies diclofenac sodium and loperamide. Meanwhile, ACME Laboratories Ltd. is another Bangladeshi medicinal institution that provided Acarbose and Diazepam for this investigation. We obtained the remaining reagents from the Department of Pharmacy at the University of Chittagong. All chemical substances and solvents utilized in this study were of analytical grade.

### 2.2 Animals for Investigations

Swiss albino mice, both male and female, weighing 20–25 g and aged 4–5 weeks, were obtained from BCSIR (Bangladesh Council of Scientific and Industrial Research) in Chittagong. The mice are kept in a controlled setting (temperature: 25°C ± 2°C, humidity: 55% ± 5%, and 12‐h cycles of light and dark) until the experiment begins (Uddin et al. 2025). During the study, albino mice were provided with a healthy diet and noncontaminated water. Animals were euthanized following the 2013 protocol and the Swiss Academy guidelines (Leary 2013). The study was conducted in accordance with the ARRIVE guidelines. The study complied with ARRIVE guidelines and was approved by the Animal Ethics Review Board (AERB), JM Medical Assistant Training School, Chittagong (Ref: AERB‐JMMATS‐2024/01/04‐(1)).

### 2.3 Sample Plant Collection and Identification

In January and February 2023, fruits of *L. coromandelica* were collected from Kutubdia, Cox's Bazar, Bangladesh. Md. Owahidul Alam, Horticulturist, Department of Botany, University of Chittagong, subsequently authenticated this. An organized herbarium was developed, and a specimen was preserved as a voucher (voucher number CUDP/47/23).

### 2.4 Methanolic Fruit Extract Preparation

The desiccated fruits were finely ground using a mechanical grinder and preserved in an airtight container. The powdered material was soaked in methanol at a solvent‐to‐material ratio of 10:1 (v/w) and kept for 72 h at room temperature (25°C ± 2°C) with intermittent shaking to ensure efficient extraction. After maceration, the mixture was first passed through a sieve and then filtered with Whatman No. 1 filter paper. The filtrate was concentrated to dryness using a rotary evaporator (Buchi R‐210) maintained at ≤ 45°C to obtain the crude extract, which was collected and stored at 4°C until use (Nahar et al. 2025; Hossain et al. 2026). The extraction yield was 17.23%.

### 2.5 Qualitative Phytochemical Screening

Standard procedures are applied for the qualitative determination of active components present in the ME (Shaikh and Patil 2020; Tyagi et al. 2017).

### 2.6 GC–MS (Gas Chromatography–Mass Spectrometry) Analysis

The analysis was performed using gas chromatography–mass spectrometry with a Clarus 690 gas chromatograph and a Clarus SQ 8 C mass spectrometer, both from PerkinElmer, CA, USA. The HP‐5MS column was applied, with a film thickness of 0.25 μm, a diameter of 0.25 mm, and a length of 30 m, where the column temperature was first maintained at 120°C for 2 min, thereafter elevated to 320°C at a rate of 10°C per minute, and sustained for 5 min. Temperatures were kept at 220°C for the injector, 180°C for the interface, and 200°C for the ion source. A 1 μL sample was injected in splitless mode, using pure helium (99.999%) as the carrier gas at a constant flow rate of 1 mL/min for 60 min. Electron ionization was utilized to assess mass in scan mode, with a mass‐to‐charge (m/z) ratio ranging from 50 to 450 amu. The ionization energy applied was 70 eV. The solvent delay was recorded as 3 min. Compounds were tentatively identified by comparing their mass spectra with reference spectra from the NIST and Wiley mass spectral libraries.

### 2.7 Determination of Total Phenolic and Flavonoid Contents

The determination of TPC and TFC in the hydroalcoholic extract of *L. coromandelica* was conducted using standardized spectrophotometric procedures with minor adjustments. TPC was determined through the Folin–Ciocalteu method. In short, 0.025 mL of extract was combined with 1.975 mL of distilled water, then reacted with 0.125 mL of Folin–Ciocalteu reagent and 0.375 mL of 20% (w/v) sodium carbonate solution. After incubation for 2 h, the absorbance was taken at 750 nm, with the reagent blank serving as the control. Calibration curves were generated using gallic acid (300–3000 μmol/L), and results were presented as mg of gallic acid equivalents (GAE) per gram of dry weight (mg GAE/g DW). The total flavonoids were quantitatively determined by using an aluminum chloride colorimetric method. According to this method, 0.25 mL of the extracts was reacted with 1 mL of distilled water, and 0.075 mL of 5% (w/v) sodium nitrite was subsequently added. After a 5‐min interval, 0.075 mL of 10% (w/v) aluminum chloride was added and allowed to react for 6 more minutes. Then, 0.5 mL of 1 M sodium hydroxide was added to the mixture and made up to 2.5 mL with distilled water. Afterward, the contents were vortexed and the absorbance was read at 510 nm relative to a blank solution. Calibration was done using rutin ranging from 100 to 1000 μmol/L, and the result was obtained in terms of mg RE/g DW (Butu et al. 2014; Dessalegn et al. 2025; Mokhtar et al. 2018).

### 2.8 Antioxidant Activity

#### 2.8.1 ABTS Free Radical Scavenging Activity

This investigation applied the methodology described by Zeng et al. (2020) where (ABTS%+) radical determined the in vitro antioxidant activity of the ME by capturing the [2,2′‐Azino‐bis(3‐ethylbenzothiazoline‐6‐sulfonic)] (ABTS%+) radical. In brief, a mixture containing 2 mM ABTS diammonium salt and 3.5 mM potassium persulfate in distilled water was incubated in the dark for 16 h to form the ABTS^+^ radical. ME between 62.5 and 500 mg/mL was mixed with 290 μL of the ABTS^+^ solution in a 96‐well plate. The mixture was incubated for 10 min. We recorded the absorbance at 750 nm, using ascorbic acid as the positive control. The percentage of ABTS free radical scavenging was calculated with the following equation (Khalid et al. 2025):

(1) $$\%\text{of}\;\text{ABTS radical scavenging}=\frac{A_{c}-A_{s}}{A_{c}}\times 100$$

Here, *A*_c_ = absorbance of control and *A*_s_ = absorbance of sample. That concentration is responsible for a 50% reduction in ABTS radical, which is used to calculate IC_50_.

#### 2.8.2 DPPH Free Radical Scavenging Activity

In this experiment, the revised version of Ismail et al. (2017). A protocol was used to determine the DPPH free radical reduction rate of the sample extract. A stock solution of 0.1 mM DPPH was prepared in methanol, and 1 mL of the extract solution containing 62.5–500 mg/mL was mixed with 5 mL of ascorbic acid at 62.5–500 mg/mL. Here, ascorbic acid is employed as a standard. The samples were incubated for 30 min in the dark setting, and their absorbance was measured at 517 nm. The percentage value for the reduction of DPPH free radicals was calculated through the following equation (Chirumamilla and Taduri 2023):

(2) $$\%\text{of DPPH radical scavenging}=\frac{A_{c}-A_{s}}{A_{c}}\times 100$$

Here, *A*_c_ = absorbance of control and *A*_s_ = absorbance of sample. That concentration is responsible for a 50% reduction in DPPH radical, which is used to calculate IC_50_.

### 2.9 Anti‐Diabetic Activity

#### 2.9.1 Evaluation of Alpha Amylase Inhibition

The methanolic fruit extract's alpha‐amylase inhibition was examined by the modified protocol of Elya et al. (2015). Acarbose and methanolic extract (12.5–100 mg/mL) were mixed with 10 mL of amylase, which had been pre‐incubated in 20 mM sodium phosphate buffer at pH 6.7. This mixture was then incubated for 5 min at 37°C. After incubation, the solution was diluted to a total volume of 2 mL with the starch solution (0.2% w/v) and then incubated again for 5 min at 37°C. Following the second incubation, 1 mL of dinitrosalicylic acid reagent was added, and the mixture was heated in a hot‐water bath. After the solution cooled for 5 min, deionized water was added. The absorbance was measured at 540 nm wavelength, and the a‐amylase inhibition was mathematically determined by the following equation (Lokapur et al. 2020):

(3) $$\text{Inhibition}\;\left(\%\right)\;\text{of}\;\alpha \;\text{amylase}=\frac{A_{c}-A_{s}}{A_{c}}\times 100$$

Here, *A*_c_ = absorbance of control, and *A*_s_ = absorbance of sample, and IC_50_ is the concentration required to inhibit 50% of α‐amylase activity.

### 2.10 Cytotoxicity Activity

#### 2.10.1 Biological Assessment of Brine Shrimp Lethality

The cytotoxic potential of the plant extracts was evaluated using the brine shrimp lethality assay (BSLA) (Mohammad, Rasel, et al. 2025). Synthetic seawater was prepared by dissolving 38 g of NaCl in 1000 mL of distilled water, with NaOH added to maintain a stable pH. Brine shrimp eggs were hatched in this synthetic seawater to produce nauplii. Test samples were prepared by serial dilution in dimethyl sulfoxide (DMSO) to yield final concentrations of 12.5, 25, 50, and 100 μg/mL. Colchicine, used as the positive control, was similarly diluted to the same concentration range, while DMSO alone served as the negative control. Healthy nauplii were counted under visual inspection at room temperature (25°C) and transferred into vials containing 5 mL of simulated seawater. Each vial was then spiked with 10 μL of the respective sample or control solution using a micropipette. After 24 h of incubation, the number of surviving nauplii in each vial was recorded, and mortality percentages were calculated to determine LC_50_ (lethal concentration for 50% mortality) values. LC_50_ was defined as the concentration that caused 50% mortality.

#### 2.10.2 Using the HeLa Cell Line

The cytotoxic effect was investigated at the Centre for Advanced Research in Sciences using their commercial services. In brief, HeLa, a human cervical cancer cell line, was cultured in DMEM (Dulbecco's Modified Eagle's Medium) supplemented with 1% penicillin–streptomycin (1:1), 0.2% gentamicin, and 10% fetal bovine serum. The HeLa cell line used in this study has the Research Resource Identifier (RRID): CVCL_0030. Cells (4.0 × 10^4^/200 μL) were placed in a 48‐well plate and incubated at 37°C, 5% CO_2_, 95% air, and 100% relative humidity. After 24 h, 50 μL of the sample was added, and the mixture was incubated for another 24 h. Cell death was assessed using trypan blue staining under an inverted light microscope, where blue‐stained cells indicated non‐viability (Tiwary et al. 2015). The following equation (Aguerd et al. 2025) expresses the percentage of viability.

(4) $$\left(\%\right)\;\text{of viability}=100\frac{\text{survival cell count}}{\text{total cell count}}$$

### 2.11 Antibacterial Property

#### 2.11.1 Agar Well Diffusion Method

The antibacterial activity of the ME was identified by applying the agar well diffusion method, which was modified from Maqbool et al.'s approach (Maqbool et al. 2020). This experiment was conducted against Gram‐positive indicator pathogens, including Bacillus, *Staphylococcus pyogenes*, and methicillin‐resistant *Staphylococcus aureus* . In contrast, Gram‐negative indicator pathogens included *Escherichia coli* , *Pseudomonas aeruginosa* , and *Pseudomonas vulgaris*. The bacteria were taken from Chittagong Medical College. A high‐temperature autoclave was used to prepare Mueller‐Hinton agar. After that, the solution was aseptically transferred to a petri dish, which was then placed under a laminar flow hood until the liquid solidified. After solidification, a thin layer of bacterial smear was applied to the media, and wells were created with a cork borer. Finally, 10 μL of each solution (extract, standard, and control) was added to the well, and the well was incubated for 24 h in an incubator to support bacterial growth. After 1 day, the zone of inhibition was measured using a ruler.

### 2.12 Anti‐Inflammatory Activity

#### 2.12.1 Carrageenan‐Induced Paw Inflammation Assay

Throughout the experiment, the mice received an intraperitoneal injection of 100 μL of thiopental sodium (50 mg/kg body weight) to induce anesthesia. According to Anyasor et al. (2019), the effects of carrageenan‐induced paw edema were investigated. Mice were randomly divided into four groups for this investigation, with each group consisting of five mice. Before the experiment, the mice had unrestricted access to water and were fasted overnight. This was the design of the experiment: Mice in Group A received (1 mL 0.9% NaCl + carrageenan induction of arthritis) known as control group; mice in Group B received (10 mg/kg body weight [b.w.] diclofenac sodium + carrageenan induction) known as standard group; and mice in Groups C and D received (200 and 400 mg/kg b.w. sample solution + carrageenan induction) known as test groups. The initial paw inflammation of each mouse was assessed using a micrometer screw gauge before treatment. After an hour of therapy, 0.1 mL of a 1% carrageenan solution was injected into the left hind paw's subplantar area to cause edema. The rise in left paw edema was then evaluated every hour for 6 h after therapy. Utilizing the following equation (Raza et al. 2025), the percent inhibition of inflammation was computed:

(5) $$\text{Inhibition of inflammation}\;\left(\%\right)=\frac{V_{c}-V_{t}}{V_{c}}\times 100$$

The average paw inflammation in the treated mice group, represented by *V*_t_, and the average paw inflammation in the control mice group, represented by *V*_c_.

### 2.13 Sedative Activity

#### 2.13.1 Open Field Assay

An open field test is performed to assess a drug's sedative potential. The open‐field setup research was conducted using the techniques described by Rauf et al. (2020). The Swiss Albino mice were divided into four groups: a positive control (0.5 mg/kg diazepam) and a negative control (saline injection), with doses of 200 and 400 mg/kg of extract administered to the test groups. The open‐field test was performed in a light‐ and sound‐controlled room. The test area was typically divided into various black‐and‐white square shapes, with the open space surrounded by a partition to prevent the subject from escaping. The wall was approximately 50 cm high. The control, standard, and sample groups of mice were orally treated and subsequently allowed to navigate freely in the open field. The number of boxes traversed by mice was recorded at 0, 30, 60, 90, and 120 min after therapy administration, during a 3‐min period (Mohammad, Mamun, et al. 2025).

#### 2.13.2 Hole Cross Method

The hole‐cross assay was applied to determine a drug's sedative properties. The hole‐cross test was conducted following the modified protocol described by Mamun, Mizan, et al. (2025). Mice were divided into four groups: two test groups received doses of 200 and 400 mg/kg of the extract, respectively; a control group received a tween solution; and a standard group received 0.5 mg/kg of diazepam. The entire study used a hole‐crossing apparatus consisting of a wooden box measuring 30 × 20 × 14 cm and featuring a partition with a circular hole in its center. Mice were placed in any compartment of the box and allowed to move freely between compartments through the circular opening. The number of holes crossed was recorded at 0, 30, 60, 90, and 120 min after the treatments, for a total of 5 time points.

### 2.14 In Silico Analysis

#### 2.14.1 Software Tools

Molecular docking studies were performed using an extensive range of software and computational tools. RCSB Protein Data Bank (rcsb.org), BIOVIA Discovery Studio 16.1, SwissPDBViewer, PyRx Virtual Screening Tools (Mamun, Rasel, et al. 2025), Open Babel GUI, PubChem, Schrödinger, and PyMOL were among the tools used (Mohammad, Chowdhury, et al. 2025). Moreover, ProTox‐3 was used to forecast the compounds' toxicity profiles (Aati et al. 2025), and SwissADME was employed for ADME (Absorption, Distribution, Metabolism, and Excretion) analysis (Mohammad, Islam, et al. 2025).

#### 2.14.2 Ligand Structure Preparation & Rationale for Selection of Molecular Targets

The GC–MS profiling for *L. coromandelica* fruit extract identified 18 components. The PubChem database provided 3D structures of these chemicals for docking. The Open Babel GUI was used to convert the compounds with available 2D structures into 3D structures in .sdf and .mol formats, respectively. Energy minimization was performed in the Swiss PDB viewer prior to molecular docking, considering key criteria such as element type, hybridization, and connectivity. After that, the ligands were converted to AutoDock Ligand format (PDBQT) for further evaluation (Hasan et al. 2025).

#### 2.14.3 Protein Preparation, Molecular Docking Analysis, and Visualization

The crystal structures of six target proteins, GABA receptor alpha1‐beta2‐gamma2 subtype (protein ID: 6X3X) was selected because it represents the predominant benzodiazepine‐sensitive receptor subtype responsible for mediating sedative–hypnotic effects in the central nervous system (Afroz et al. 2025). The Pancreatic ATP‐sensitive potassium channel (protein ID: 5YW7) was selected due to its key role in insulin secretion and glucose regulation (Patle et al. 2024; Sultana, Shikder, et al. 2025; Sultana, Hasan, et al. 2025). The Caspase‐3 (protein ID: 1NMS) was selected due to its central role in apoptosis, making it a key target for evaluating cytotoxic activity (Fallon Adido et al. 2023). The Human myeloperoxidase‐thiocyanate complex (protein ID: 1DNU) was selected due to its role in oxidative stress and reactive species generation, making it relevant for antioxidant evaluation (Abera et al. 2024). The Human COX‐1 crystal structure (protein ID: 6Y3C) was selected due to its key role in prostaglandin synthesis, making it a relevant target for anti‐inflammatory activity (Islam et al. 2024). The GGDEF‐EAL domain‐containing c‐di‐GMP receptor FimX (protein ID: 3HVA) was selected due to its role in bacterial signaling and biofilm regulation, making it relevant for antibacterial activity. All protein structures were retrieved from the RCSB PDB and prepared using BIOVIA Discovery Studio, where water molecules were removed, polar hydrogens were added, and structural inconsistencies were corrected. The binding site integrity was further validated using PyMOL. (Hossain et al. 2025; Mamun, Mizan, et al. 2026).

Ligands and reference drugs were converted to pdbqt in PyRx, with partial charges and rotatable bonds assigned; AutoDock Tools added hydrogens and defined torsions. Energy minimization was done in Swiss‐PDB Viewer. A grid box with a spacing of 0.306 Å and dimensions of X: 36.112 Å, Y: 33.0472 Å, and Z: 12.1999 Å was generated using AutoGrid to define the docking search space around the active site of the target protein. Flexible docking was performed using AutoDock Vina, analyzed via PyRx, and interactions visualized in BIOVIA Discovery Studio to assess hydrogen bonds, hydrophobic contacts, π–π stacking, and other non‐covalent interactions (Mamun, Rasel, et al. 2026).

### 2.15 Statistical Data Assessment

Data were analyzed using SPSS version 25 (ANOVA with Dunnett's post hoc; *p < 0.05, **p < 0.01, ***p < 0.001) and expressed as Mean ± SEM. Chemical structures were drawn in “ChemDraw Ultra 12.0.1,” while IC_50_ and LC_50_ values were calculated with GraphPad Prism (version 8.0.1) and Microsoft Excel 2024.

## 3 Results

### 3.1 Qualitative Phytochemical Analysis

This result expressed the presence of various phytochemicals in the ME (Table 1).

**TABLE 1:** Qualitative phytochemical screening of the ME.

Phytochemicals	Specific test	Test result
Alkaloids	Mayer's test	+
	Hager test	+
	Wagner test	+
Flavonoids	Alkaline reagent test	+
Saponins	Foam test	+
Tannins	Gelatin test	+
Phenolic compound	Ferric chloride test	+
Glycoside	Liebermann's test	+
Carbohydrate	Benedict's test	+
Reducing sugar	Fehling's test	−
Protein and amino acid	Biuret test	+
	Ninhydrin test	+
Acidic compound	Litmus test	+
	Ferric chloride test	+
Phytosterol	Liebermann–Burchard test	+
	Salkowski test	+
Steroids and terpenes	Liebermann–Burchard test	+

*Note:* (+) sign represents the presence of phytochemicals, where (−) sign represents the absence of phytochemicals.

### 3.2 GC–MS Result

**FIGURE 1:**
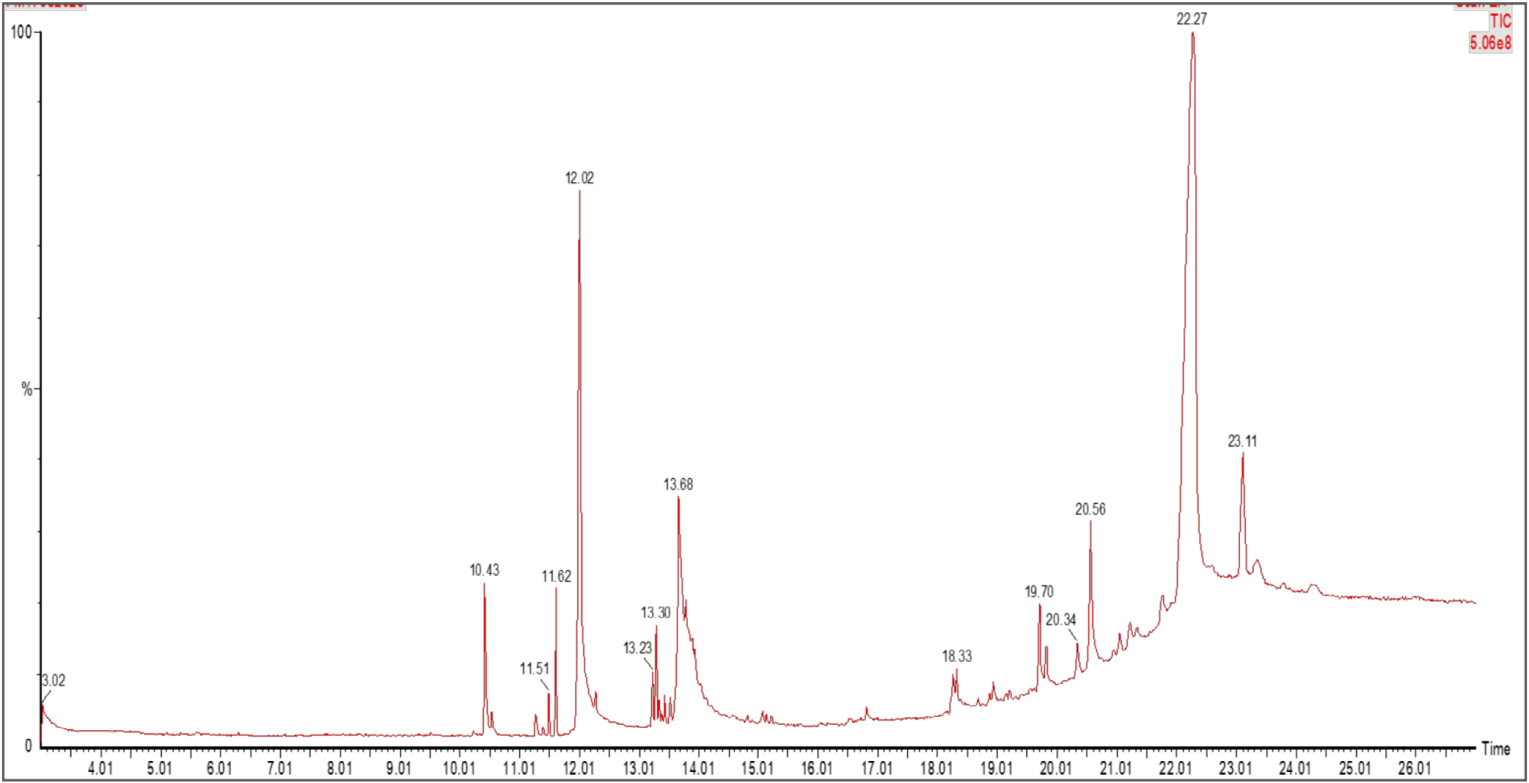
Graphical presentation of GC–MS findings of the ME.

GC–MS profiling of ME yielded tentative identification of 18 phytochemical constituents (Table 2, Figure 1). The following classes were identified: fatty acids and their esters (e.g., methyl 11‐hexadecenoate, hexadecanoic acid [palmitic acid], and hexadecanoic acid ethyl ester), long‐chain ketones/aldehydes (e.g., cyclopropaneoctanal and 2‐octyl‐), phenolic derivatives ((Z)‐3‐(heptadec‐10‐en‐1‐yl)phenol), sterols (γ‐sitosterol), amide derivatives (trans‐13‐docosenamide/erucamide), and a few other terpenoid/alkyl derivatives. The diverse range of phytochemical constituents detected by GC–MS profiling highlights the complex chemical composition of ME, which may contribute to its potential physiological functions. Table 2 presents a concise list of the discovered compounds, including their retention periods and chemical formulas. The GC–MS‐detected constituents correspond well with previously described bioactive chemical classes, wherein fatty acids/esters are linked to antioxidant and anti‐inflammatory activities, phenolic derivatives with antimicrobial (Rouvier et al. 2024) and cytoprotection, and phytosterols (γ‐sitosterol) with anti‐inflammatory (Pauloi et al. 2024) and metabolic regulatory functions. Additionally, scaffolds such as dodecyl isothiocyanate and erucamide m/mass fractions that have not been previously well‐studied in this context were present, suggesting that this extract is a valuable source of novel lead molecules for mechanism‐driven drug discovery.

**TABLE 2:** Phytochemical composition of ME.

No.	Compound name	Molecular weight	Retention time	Molecular formula	% of area
1	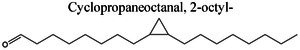	280	10.43	C_19_H_36_O	2.09
2	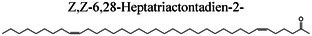	530	10.55	C_37_H_70_O	0.15
3	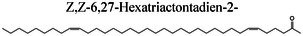	516	11.28	C_36_H_68_O	0.22
4	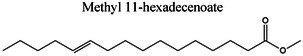	268	11.51	C_17_H_32_O_2_	0.36
5	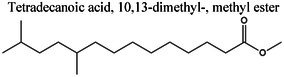	270	11.62	C_17_H_34_O_2_	2.54
6	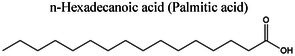	256	12.02	C_16_H_32_O_2_	10.38
7	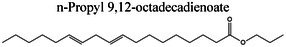	322	13.23	C_21_H_38_O_2_	0.39
8	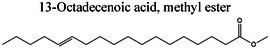	296	13.30	C_19_H_36_O_2_	0.55
9	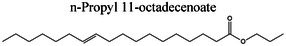	324	13.68	C_21_H_40_O_2_	4.23
10	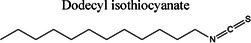	227	16.82	C_13_H_25_NS	0.21
11	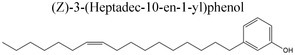	330	18.33	C_23_H_38_O	0.90
12	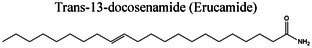	337	18.93	C_22_H_43_ON	0.21
13	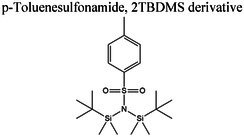	399	19.70	C_19_H_37_O_2_NSSi	2.24
	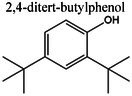	206	19.83	C_14_H_22_O	0.87
15	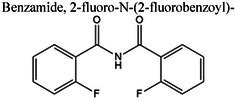	415	20.34	C_20_H_12_O_2_NBrF	1.01
16	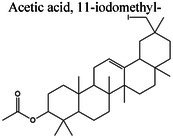	594	22.27	C_32_H_51_O_2_I	65.56
17	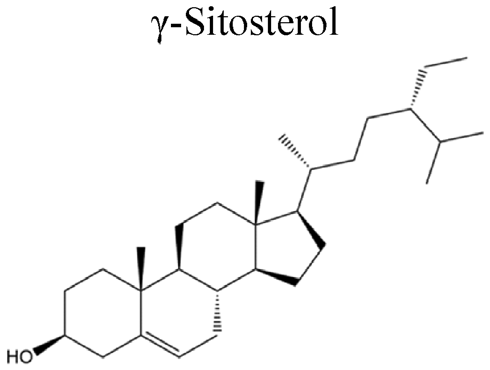	414	23.11	C_29_H_50_O	0.47
18	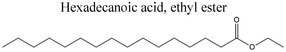	284	12.28	C_18_H_36_O_2_	0.33

### 3.3 Total Phenolic and Flavonoid Content

The findings revealed that ME possesses substantial levels of total phenolic content (TPC) and total flavonoid content (TFC) (Table 3).

**TABLE 3:** Total phenolic and flavonoid content of ME.

TPC of ME mg QE/g	TFC of ME mg QE/g
35 ± 1.03 mg OE/g	11.57 ± 0.37 mg QE/g

### 3.4 Evaluation of Antioxidant Activity

#### 3.4.1 ABTS Radical Scavenging Assay

**FIGURE 2:**
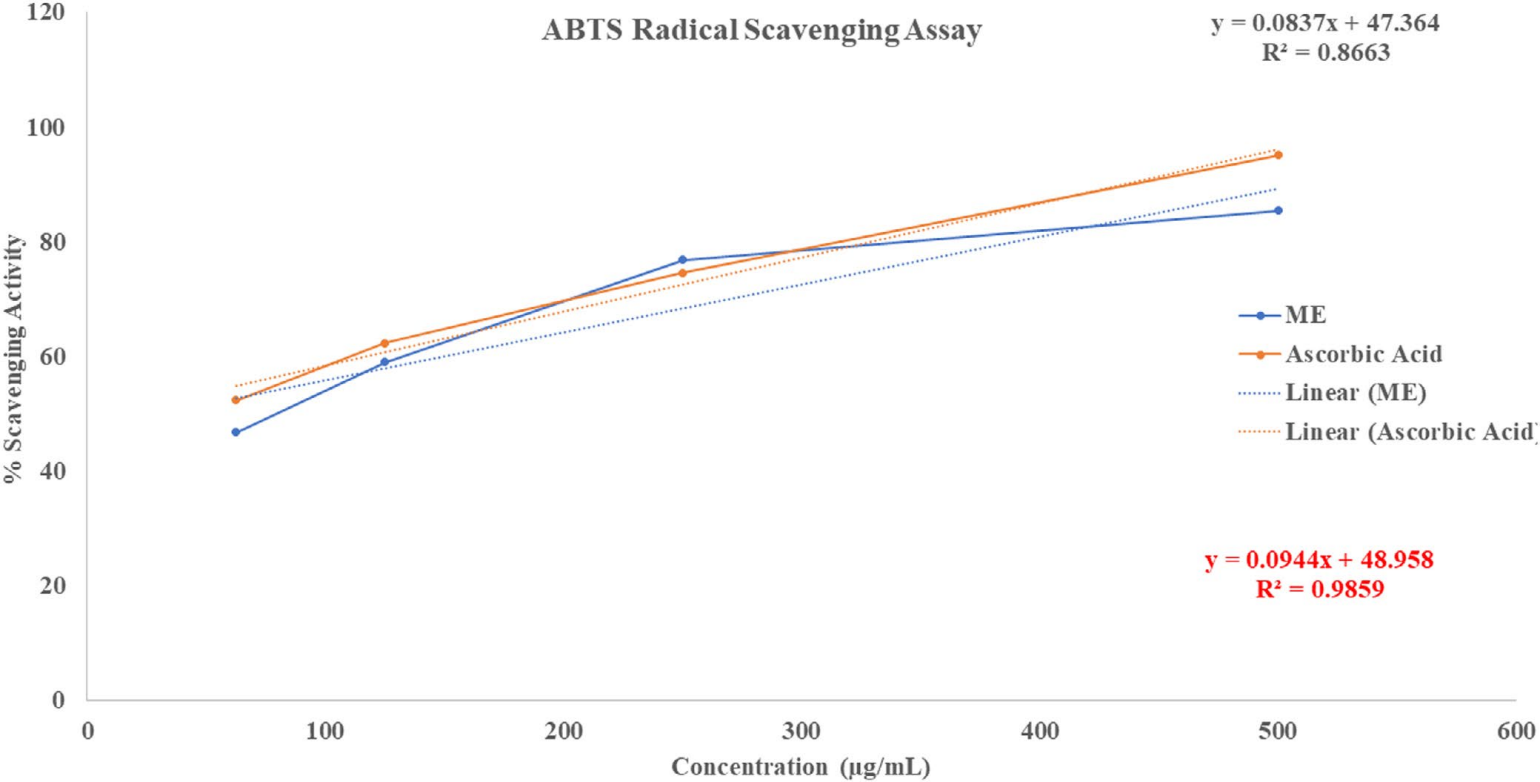
Evaluation of antioxidant activity of ME through ABTS radical scavenging assay.

The ABTS free radical scavenging method is used to demonstrate the antioxidant capability of the ME, as shown in Figure 2. The antioxidant effect of ME increased with concentration, as evidenced by free radical scavenging activities of 46.78%, 58.96%, 76.78%, and 85.43% against ABTS (2,2′‐azino‐bis (3‐ethylbenzothiazoline‐6‐sulfonic acid)) at different concentrations. The extract's cumulative IC_50_ value was 31.49 μg/mL, while conventional ascorbic acid's IC_50_ value was 11.04 μg/mL. The extract exhibited strong free radical scavenging activity; however, its activity was slightly lower than that of ascorbic acid, suggesting it could be used as a moderate source of natural antioxidants.

#### 3.4.2 DPPH Radical Scavenging Activity

**FIGURE 3:**
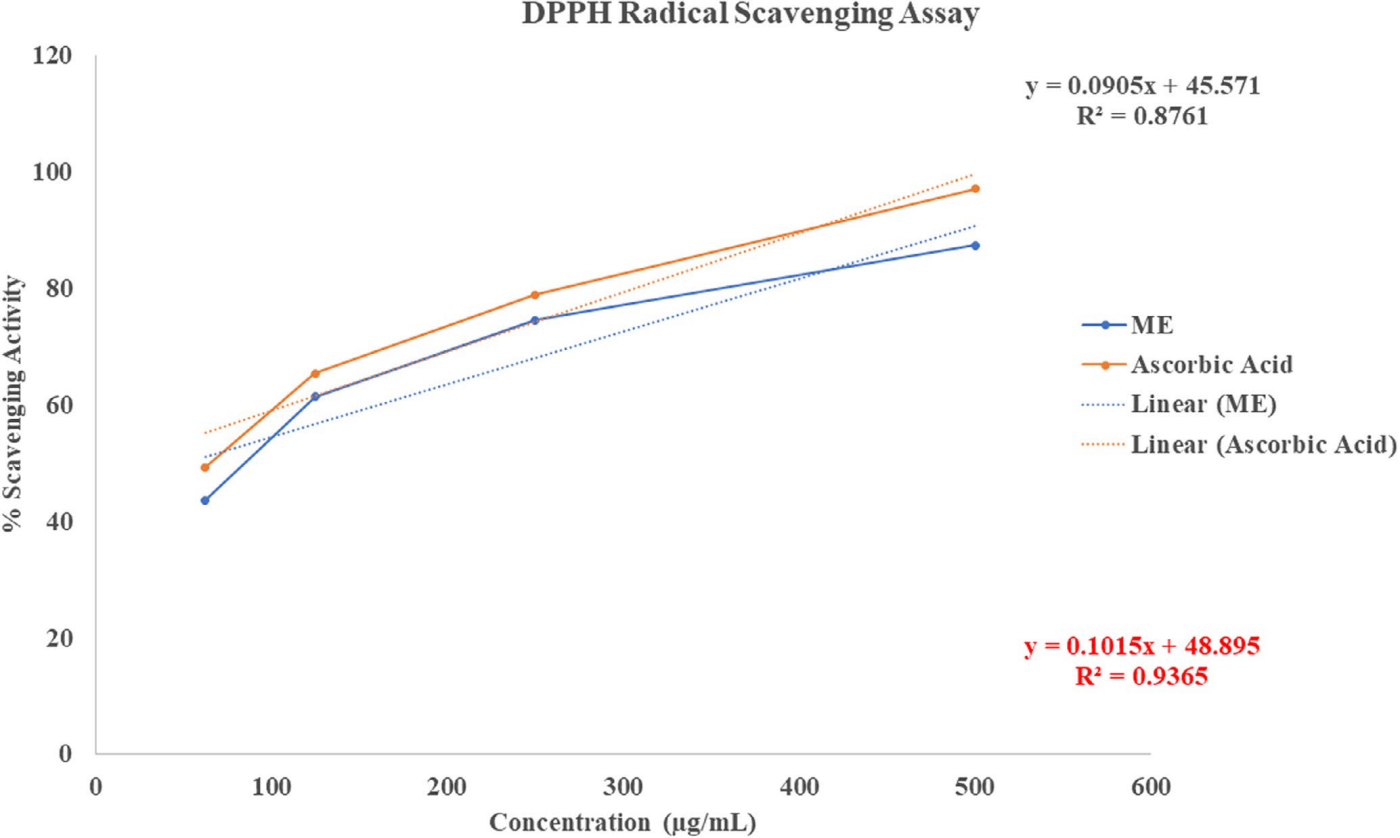
Evaluation of antioxidant activity of ME through DPPH radical scavenging assay.

Figure 3 shows the potential antioxidant activities of ME, with 43.67%, 61.45%, 74.59%, and 87.45% inhibition at different concentrations, as measured by the DPPH (2,2‐diphenyl‐1‐picrylhydrazyl) free radical degradation, indicating a concentration‐dependent increase in antioxidant activity. Additionally, the findings showed that ME's antioxidant capacity is comparable to that of ascorbic acid, a well‐known antioxidant. The calculated IC_50_ value of the extract against DPPH was 48.94 μg/mL, reflecting its effectiveness in scavenging free radicals; in contrast, ascorbic acid had an IC_50_ value of 10.89 μg/mL. The experiment highlights that, while the extract has significant antioxidant activity, it is less effective than ascorbic acid at scavenging DPPH free radicals, as evidenced by the higher IC_50_ value.

### 3.5 Evaluation of Antidiabetic Activity

#### 3.5.1 Alpha Amylase Inhibition Assay

**FIGURE 4:**
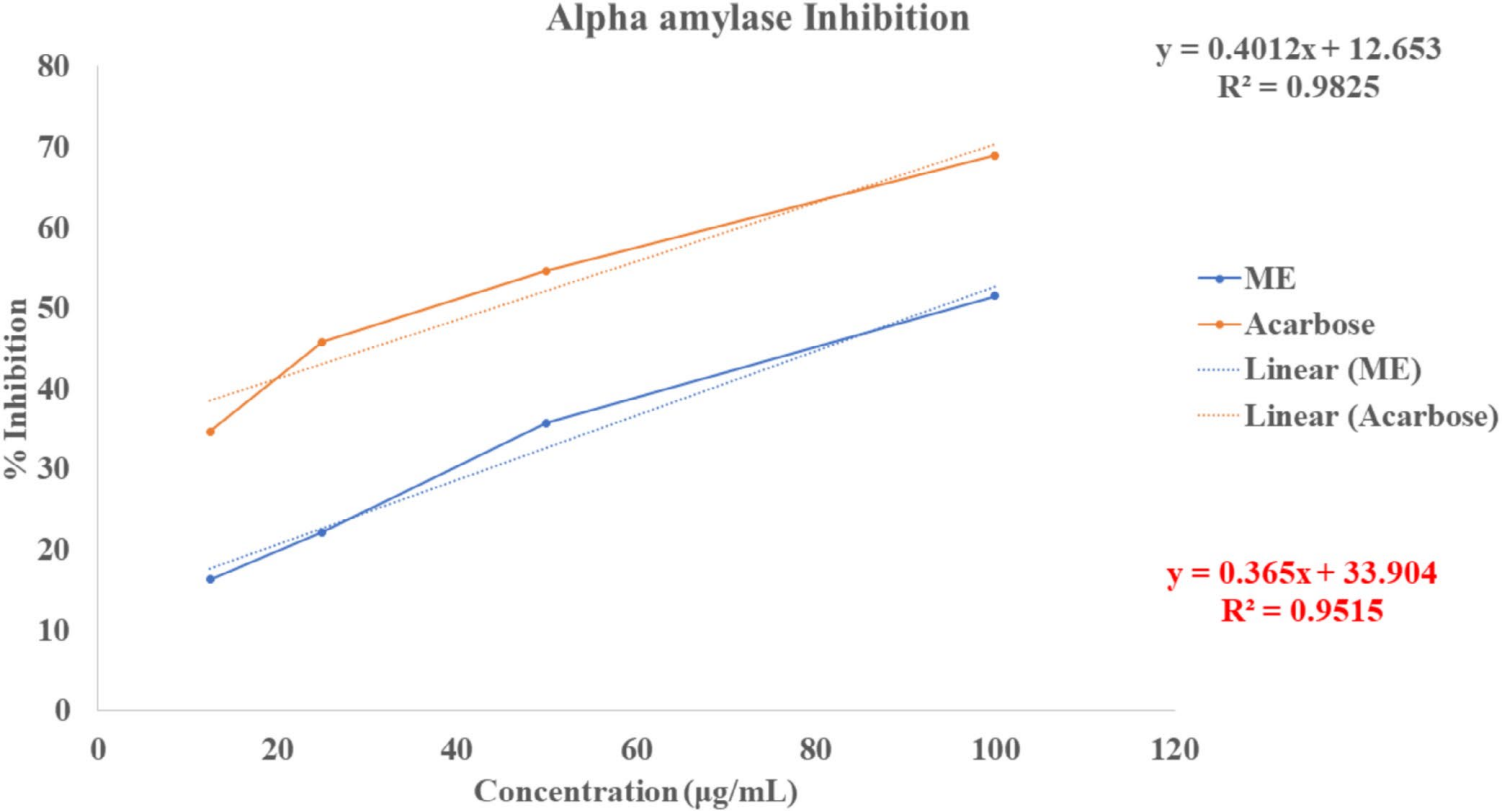
Evaluation of antidiabetic activity of ME through alpha amylase inhibition assay.

To demonstrate the antidiabetic activity of ME, this experiment quantified alpha‐amylase inhibition at various concentrations. At concentrations of 12.5–100 μg/mL, we observed a dose‐dependent increase in the % inhibition of α‐amylase from 16.33% ± 1.08% to 51.53% ± 1.24% (Figure 4). The estimated IC_50_ for ME was 93.09 μg/mL, indicating that ME exhibited potent antidiabetic activity compared with standard Acarbose's IC_50_ value (44.10 μg/mL). The results show that ME contains natural compounds with therapeutic activity, producing a strong antidiabetic effect.

### 3.6 Evaluation of Cytotoxic Activity

#### 3.6.1 Brine Shrimp Lethality Assay

**FIGURE 5:**
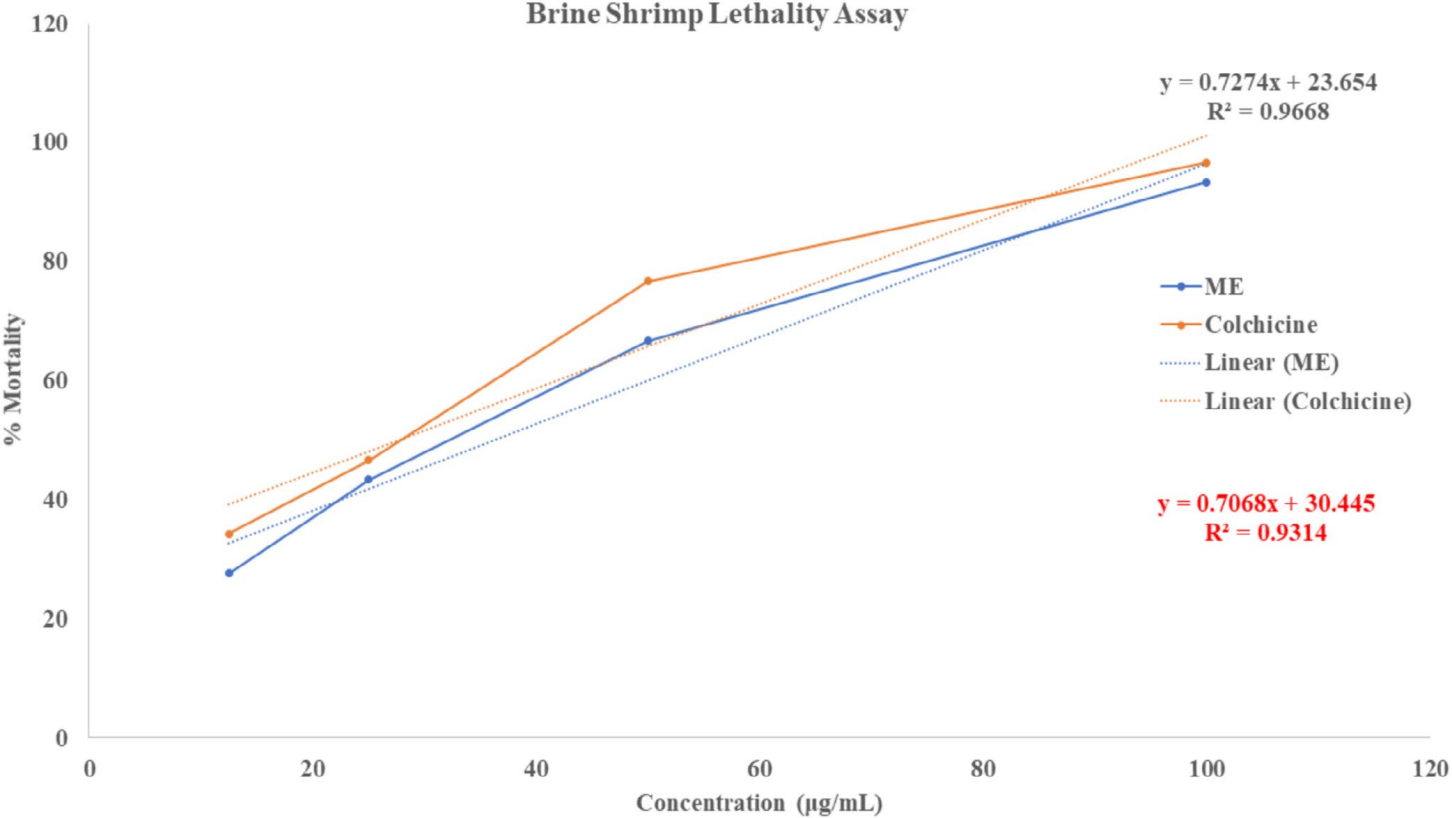
Representing the cytotoxic activity of ME using the brine shrimp lethality assay.

The brine shrimp lethality test was performed to evaluate the cytotoxicity of ME. After analyzing the raw data, the final result represented the mortality percentage of shrimp in different concentrations. The extract showed a dose‐dependent mortality rate, ranging from 27.67 ± 0.33 to 93.33 ± 0.33. At the same time, the % mortality of the standard drug, colchicine, ranged from 34.30 ± 0.58 to 96.67 ± 0.33 at concentrations of 12.5–100 μg/mL (Figure 5). Based on the results, ME shows strong cytotoxicity, with an LC_50_ of 36.22 μg/mL. In comparison, the LC_50_ value for the standard drug, colchicine, was 27.57 μg/mL.

#### 3.6.2 Viability of HeLa Cells by Trypan Blue Dye Assay

**FIGURE 6:**
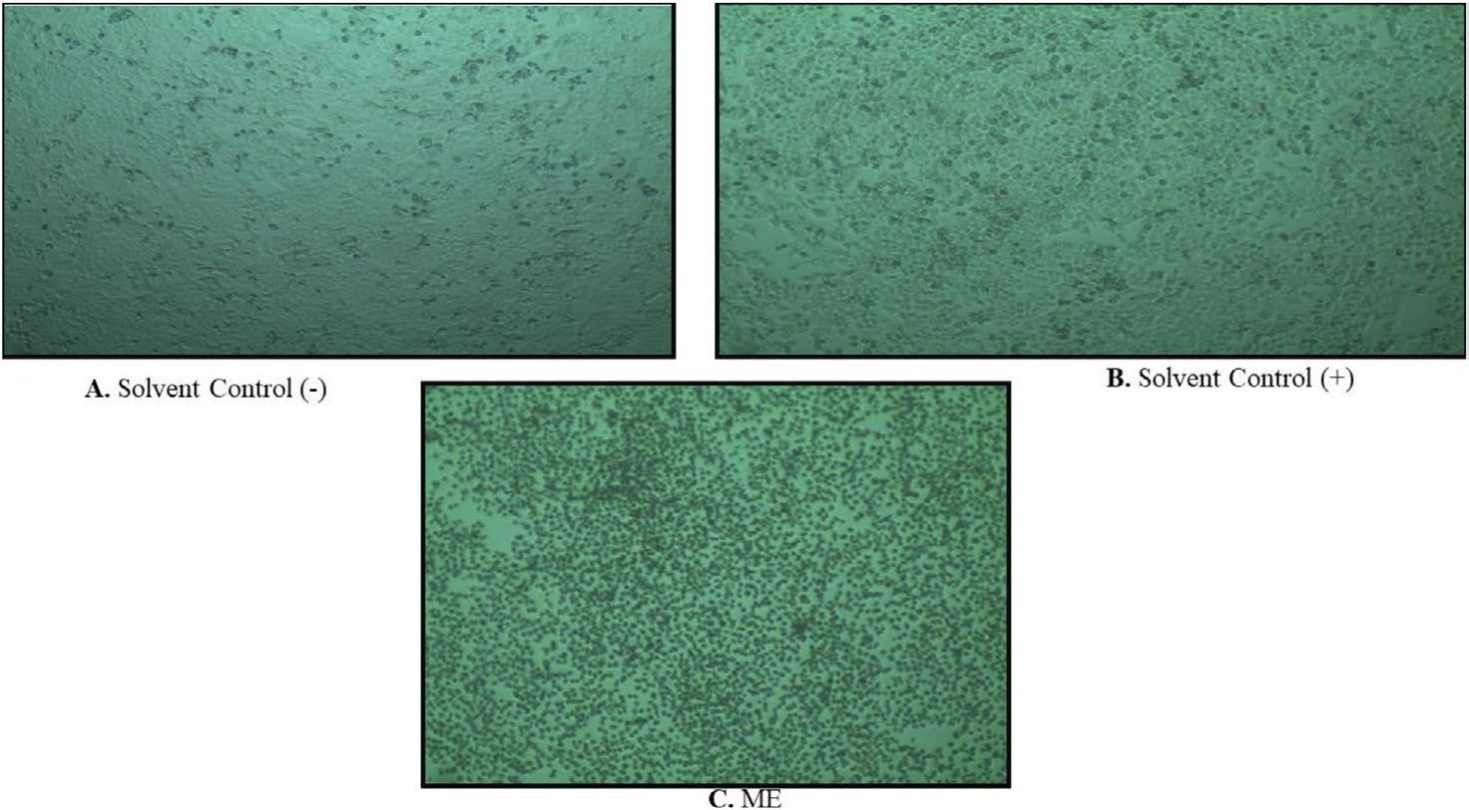
Evaluation of the cytotoxic activity of ME against the HeLa cell line using the MTT assay. Representative microscopic images show (A) negative solvent control, (B) positive solvent control, and (C) ME‐treated cells.

The Trypan Blue Dye Assay was performed to assess the cytotoxicity of ME. Under an inverted light, a significant change in cell viability was observed. Less than 5% of the HeLa cells were in the microscopic healthy plate that was treated with 50 μL ME (Figure 6, Table 4). In the meantime, 100% of cells survived in the negative control, whereas more than 95% survived in the positive control. Comparing cell viability with positive and negative controls, we can conclude that *L. coromandelica* fruit extract exhibits strong cytotoxicity against the human cervical carcinoma cell line.

**TABLE 4:** Cell viability of ME.

Sample	Survival of cells (Hella)
Solvent−	100%
Solvent+	> 95%
ME	< 5%

### 3.7 In Vitro Antibacterial Activity

#### 3.7.1 Agar Well Diffusion Assay

**FIGURE 7:**
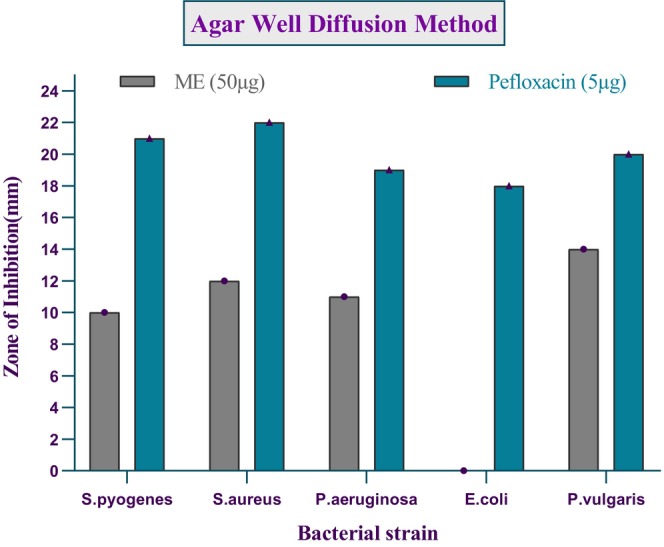
Evaluation of antibacterial activity of ME against bacterial pathogens.

In this experiment, we demonstrated ME's antibacterial activity by assessing its ability to inhibit bacterial growth or reproduction, as evidenced by the zone of inhibition around the wells. Figure 7 shows the zones of inhibition of ME and the standard Pefloxacin against selected bacterial species. This investigation revealed that the ME extract exhibited a moderate zone of inhibition, measuring 0–14 mm. In contrast, the zone of inhibition for Pefloxacin was estimated at 18 to 22 mm. The results indicate that ME contains some chemical compounds that may act against certain gram‐positive and gram‐negative bacteria, but they are not as potent as Pefloxacin.

### 3.8 In Vivo Anti‐Inflammatory Activity

#### 3.8.1 Carrageenan‐Induced Paw Edema Assay

**FIGURE 8:**
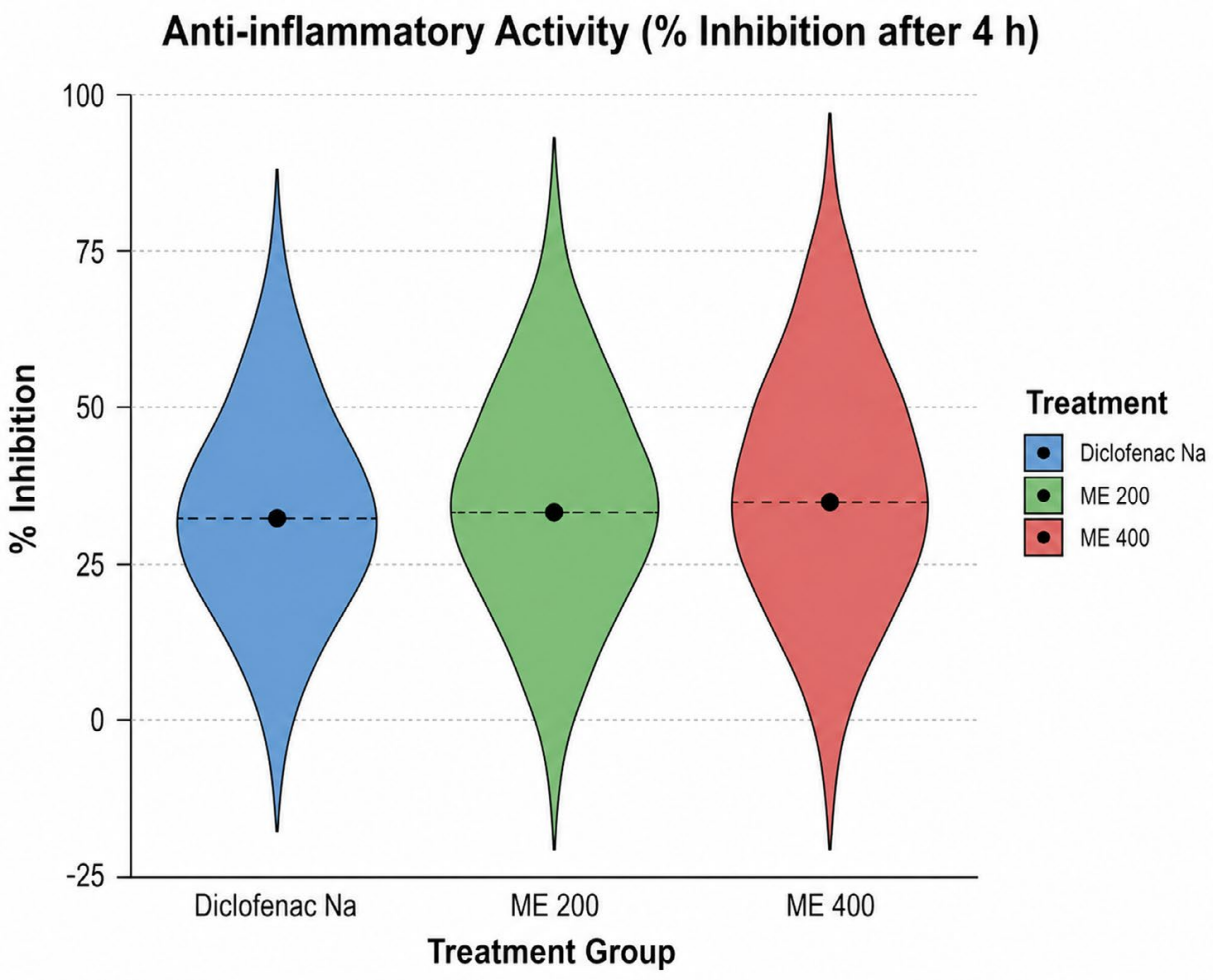
Violin plot and time‐course analysis of paw‐edema inhibition showing dose‐dependent anti‐inflammatory activity of ME compared with diclofenac in mice.

The anti‐inflammatory effect of ME was evaluated using the carrageenan‐induced paw edema assay (Table 5). Control‐treated mice showed increased paw thickness (4.14 ± 0.04 to 4.36 ± 0.05 mm at 2 h), with only partial reduction by 4 h (4.24 ± 0.04 mm), while diclofenac and ME‐treated groups showed significant edema reduction. At 4 h, paw thickness decreased to 2.98 ± 0.06 mm (standard), 2.88 ± 0.08 mm (ME 200 mg/kg), and 2.80 ± 0.10 mm (ME 400 mg/kg). ME 400 exhibited the highest inhibition of edema (33.97%), surpassing diclofenac (29.72%) and ME 200 (32.08%). Results indicate that ME possesses dose‐dependent anti‐inflammatory activity, with the 400 mg/kg dose showing greater efficacy than the standard drug. In the violin plot, the distribution of paw edema inhibition at 4 h is shown, indicating highly anti‐inflammatory activity of both ME (200 and 400 mg/kg) in a dose‐dependent manner relative to diclofenac, with maximum median inhibition observed with ME 400. For clarity, data on paw edema (Table 5) were plotted as a time‐course curve (0–4 h), and the analysis of data in Table 5 is shown in Figure 8, presented as mean ± SD with one‐way ANOVA followed by post hoc multiple‐comparison testing, with significance set at *p* < 0.05.

**TABLE 5:** Data obtained from the carrageenan‐induced paw edema test.

Treatment group	Mean (paw edema thickness) ± SEM						% inhibition after 4 h
	Before carrageenan induction	0 h	1 h	2 h	3 h	4 h	
1% Tween 80 (Control)	2.62 ± 0.06	4.14 ± 0.04	4.20 ± 0.04	4.36 ± 0.05	4.32 ± 0.07	4.24 ± 0.04	N/A
Diclofenac Na	2.86 ± 0.05	4.10 ± 0.08	3.72 ± 0.04***	3.36 ± 0.075***	3.08 ± 0.06***	2.98 ± 0.06***	29.72%
ME 200	2.76 ± 0.08	4.04 ± 0.05	3.54 ± 0.07***	3.36 ± 0.07***	3.10 ± 0.08 ***	2.88 ± 0.08***	32.08%
ME 400	2.72 ± 0.07	4.12 ± 0.05	3.74 ± 0.07**	3.40 ± 0.15**	3.08 ± 0.15 ***	2.80 ± 0.10***	33.96%

*Note:* **p < 0.01, ***p < 0.001 indicate statistically significant differences compared to the control group.

### 3.9 In Vivo Sedative Activity

#### 3.9.1 Open Field Test

**FIGURE 9:**
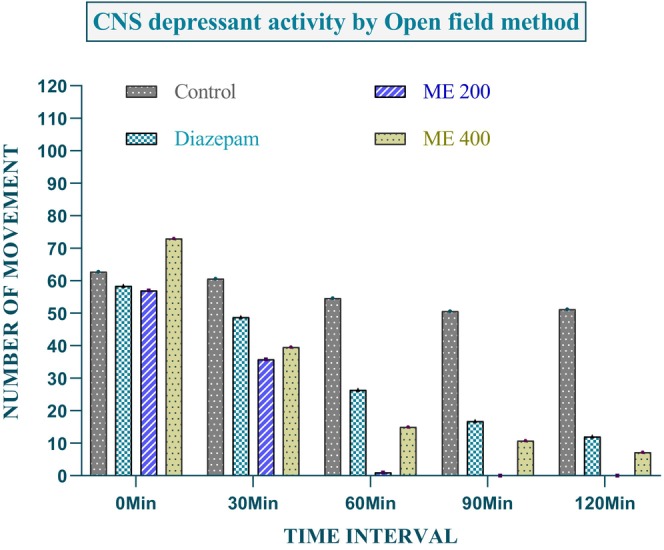
Evaluation of sedative activity of ME using the open field method.

The sedative‐hypnotic effects of ME were evaluated using the open field test (Figure 9). Both ME (200 and 400 mg/kg) and diazepam significantly reduced locomotor activity compared to the control. Diazepam decreased movements from 58.4 ± 6.32 to 12.0 ± 1.0 over 120 min (*p* < 0.001). ME at 200 mg/kg reduced activity from 57.0 ± 6.12 to complete immobility (0 ± 0), while the 400 mg/kg dose decreased movements from 73.0 ± 2.88 to 11.2 ± 5.32 (*p* < 0.001), showing dose‐dependent CNS depression. Notably, ME at 200 mg/kg induced complete immobility, suggesting more pronounced sedative effects than diazepam.

#### 3.9.2 Hole Cross Test

The hole‐cross test was used to evaluate the sedative‐hypnotic activity of ME over a 120‐min period (Table 6). ME (200 and 400 mg/kg) and diazepam significantly reduced locomotor activity compared to the control. The control group showed minimal change (9.6 ± 0.75 to 8.8 ± 0.58 movements). In contrast, diazepam reduced movements from 8.8 ± 0.58 to 1.0 ± 0.32 (*p* < 0.001). ME 200 decreased activity from 10.4 ± 2.25 to 1.0 ± 0.55, while ME 400 showed the most significant reduction—from 8.4 ± 1.54 to 0.4 ± 0.24 (*p* < 0.001). The profound decrease in locomotion indicates strong CNS depressant effects. These results suggest that ME contains bioactive compounds with potent sedative‐hypnotic activity, potentially offering a natural alternative to diazepam in sleep modulation.

**TABLE 6:** Data obtained from the hole cross experiment.

Treatment group	Dose and route	Number of movements (mean ± SEM)				
		0 min	30 min	60 min	90 min	120 min
1% Tween 80 (control)	10 mL/kg; p.o	9.6 ± 0.75	10 ± 0.7	9.6 ± 0.92	9.4 ± 0.74	8.8 ± 0.58
Diazepam	10 mg/kg; p.o	8.8 ± 0.58	5.0 ± 0.71**	3.4 ± 1.08**	2.4 ± 0.25**	1.0 ± 0.32***
ME	200 mg/kg; p.o	10.4 ± 2.25	3.6 ± 1.08**	2.8 ± 1.11**	1.2 ± 0.37***	1.0 ± 0.55***
ME	400 mg/kg; p.o	8.4 ± 1.54	4.8 ± 1.66*	3.4 ± 1.40**	0.8 ± 0.2**	0.4 ± 0.24***

*Note:* **p < 0.01, ***p < 0.001 indicate statistically significant differences between groups.

### 3.10 In Silico Study

#### 3.10.1 Docking‐Based Assessment of Lead Phytochemicals

**FIGURE 10:**
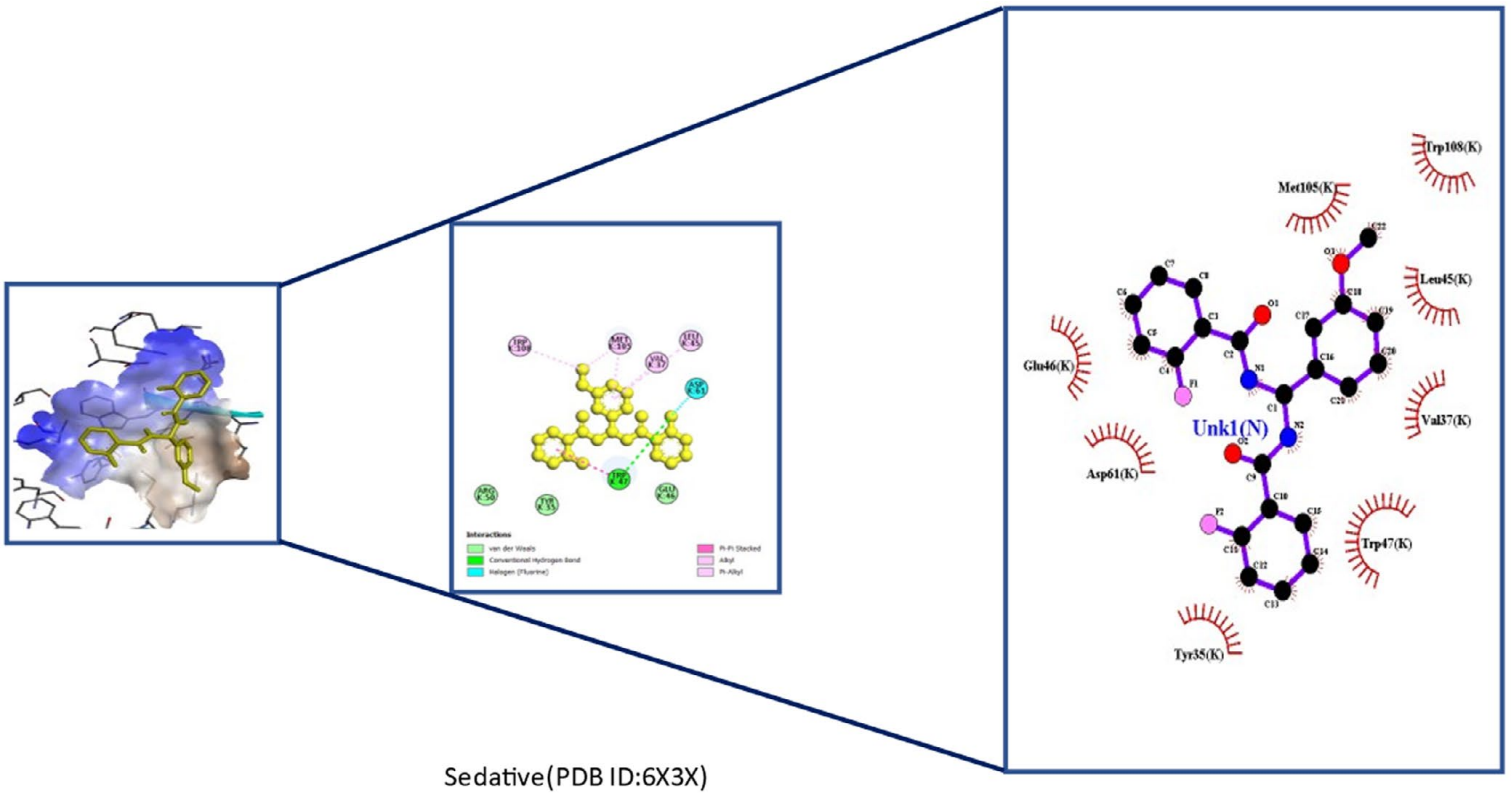
3D and 2D binding types of 2‐fluoro‐N‐[[(2‐fluorobenzoyl) amino]‐(3‐methoxyphenyl)methyl]benzamide binding with specific amino acids of sedative protein (PID: 6X3X).

**FIGURE 11:**
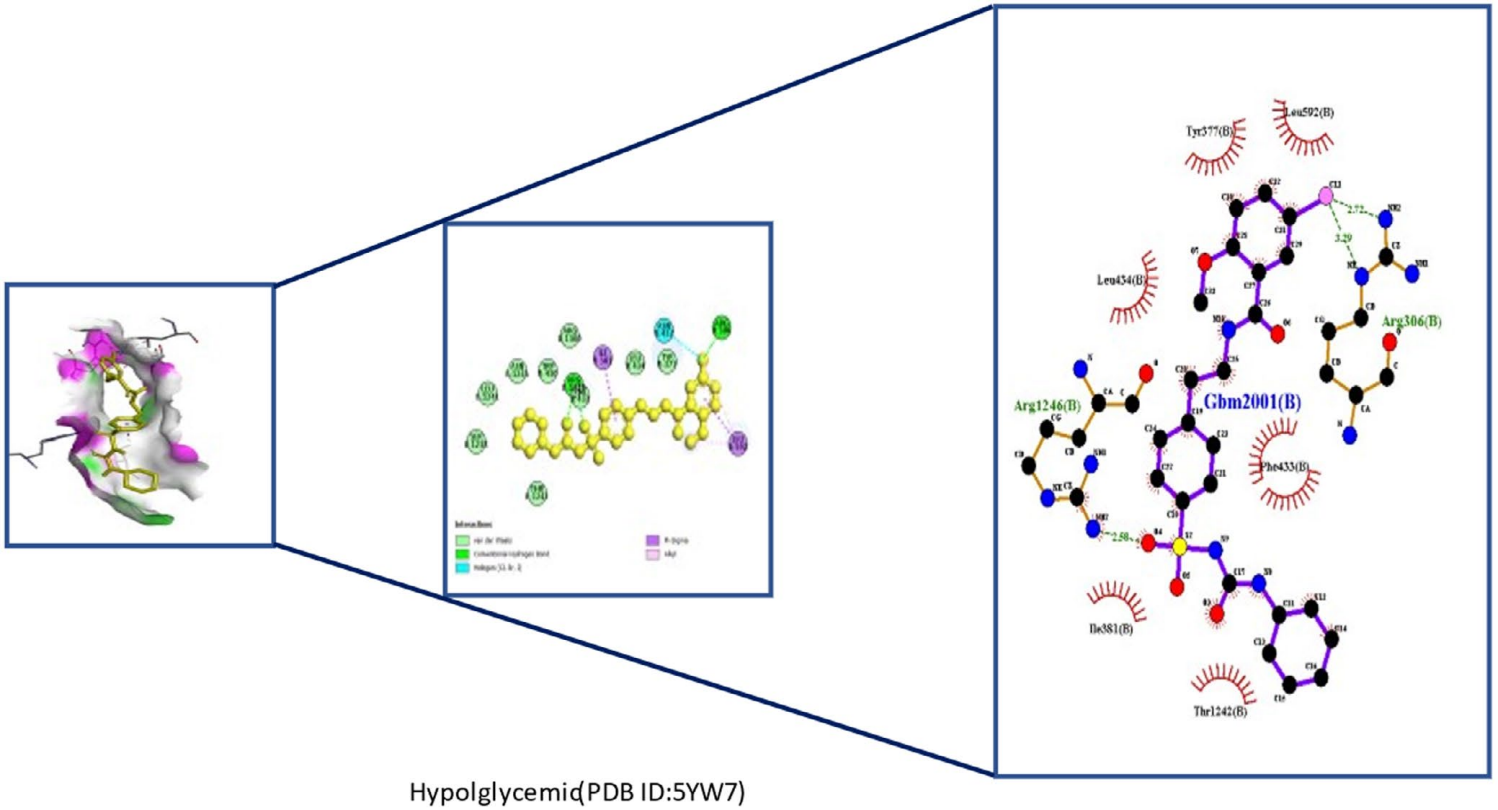
ligplot, 3D and 2D binding types of 2‐fluoro‐N‐[[(2‐fluorobenzoyl) amino]‐(3‐methoxyphenyl)methyl]benzamide binding with specific amino acids of antidiabetic protein (PID: 5YW7).

**FIGURE 12:**
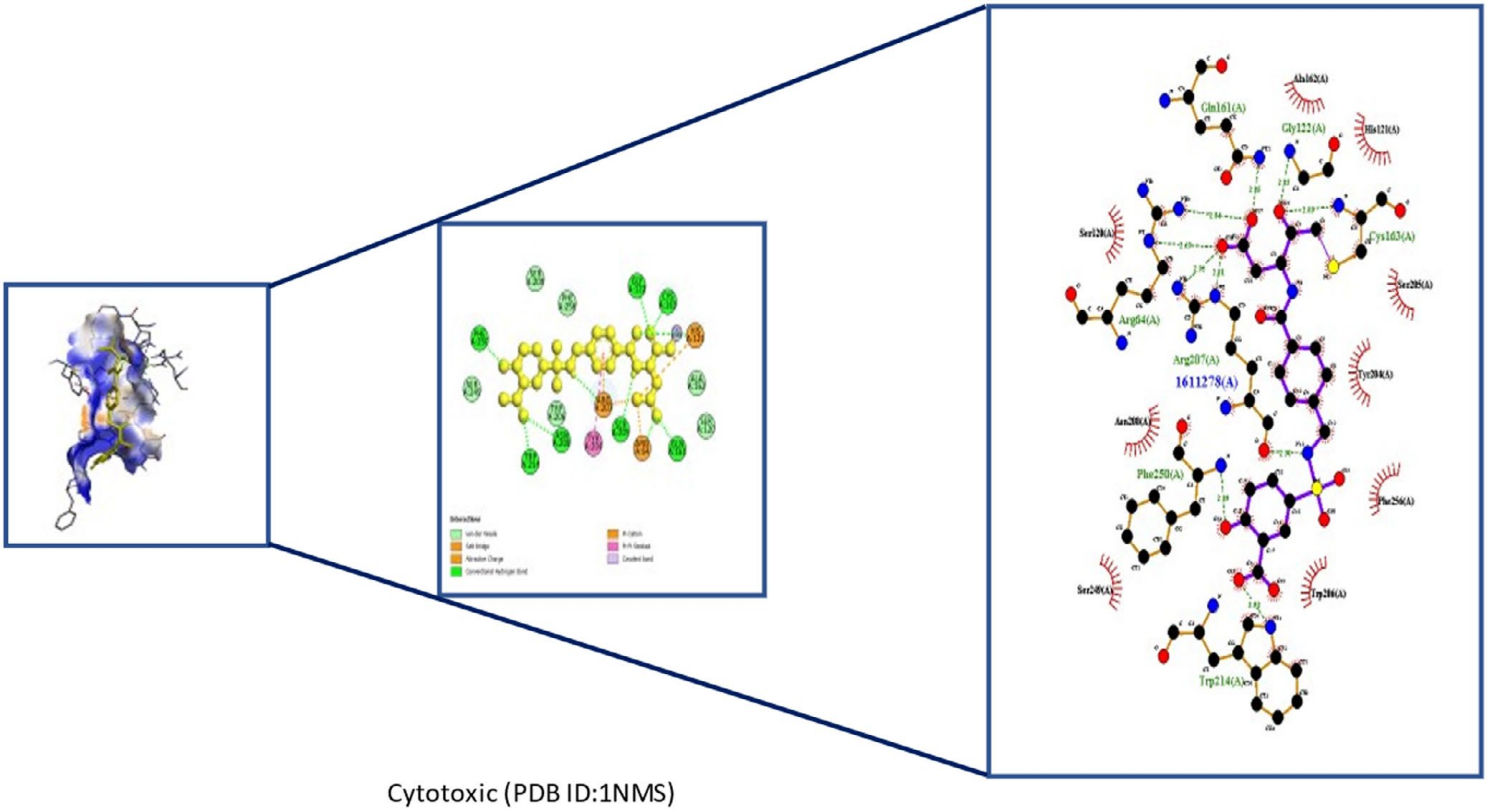
Ligplot, 3D and 2D binding types of Tetradecanoic acid, 10,13‐dimethyl‐, methyl ester binding with specific amino acids of cytotoxic protein (PID:1NMS).

**FIGURE 13:**
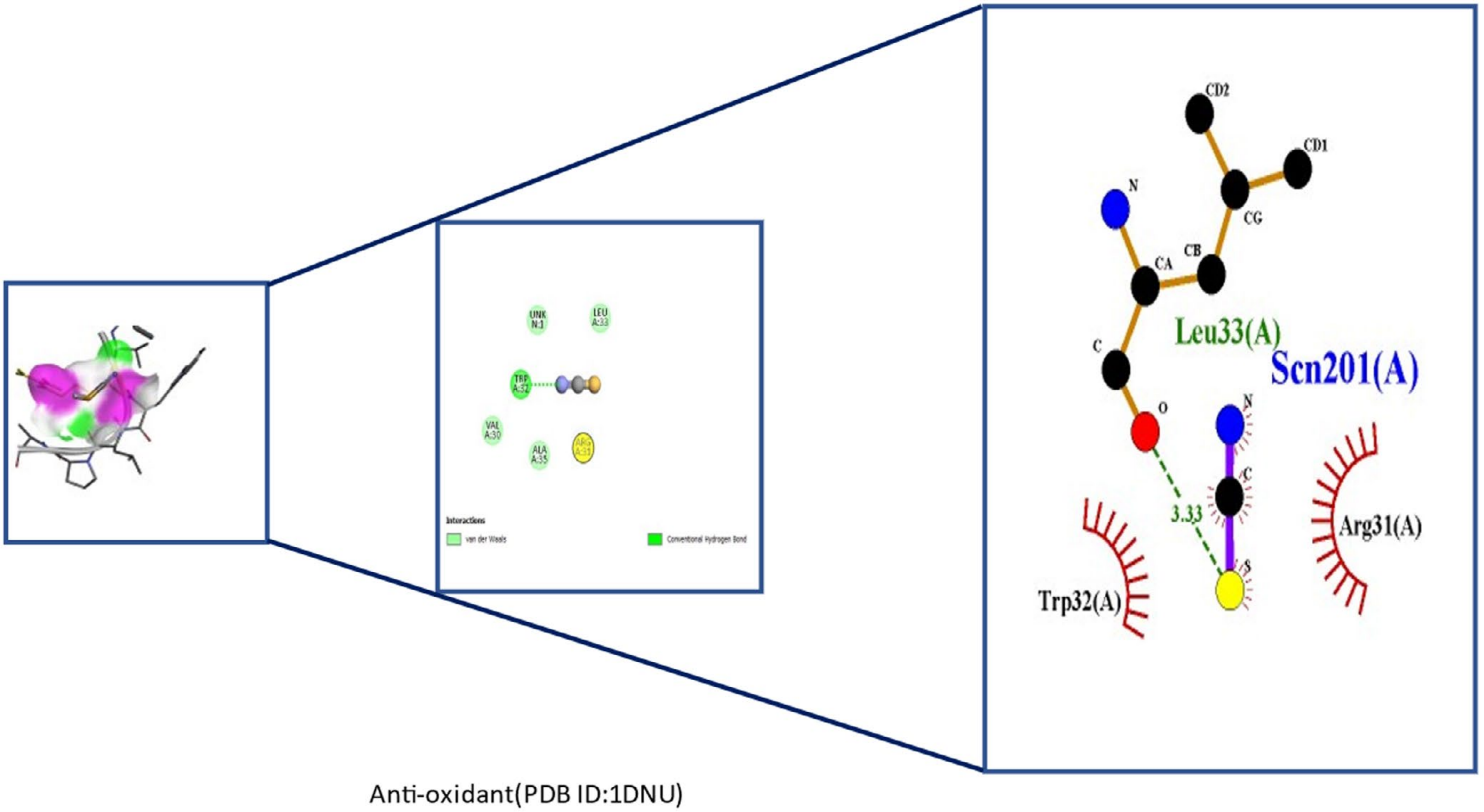
Ligplot, 3D, and 2D binding types of 3‐[(Z)‐heptadec‐10‐enyl] phenol binding with specific amino acids of the anti‐oxidant protein (PID: 1DNU).

**FIGURE 14:**
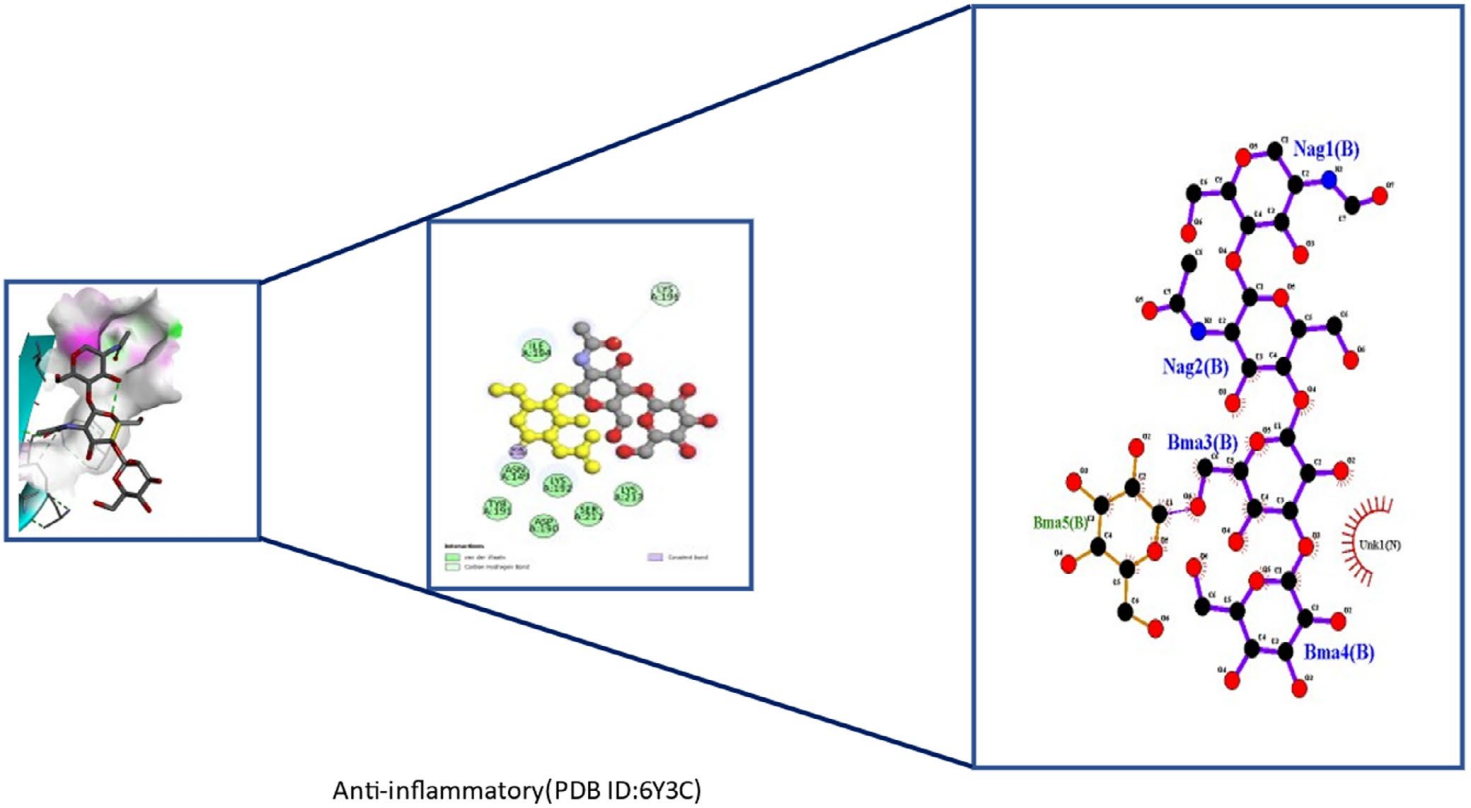
Ligplot, 3D, and 2D binding types of 8‐(2‐octylcyclopropyl)octanal binding with specific amino acids of the anti‐inflammatory protein (PID: 6Y3C).

**FIGURE 15:**
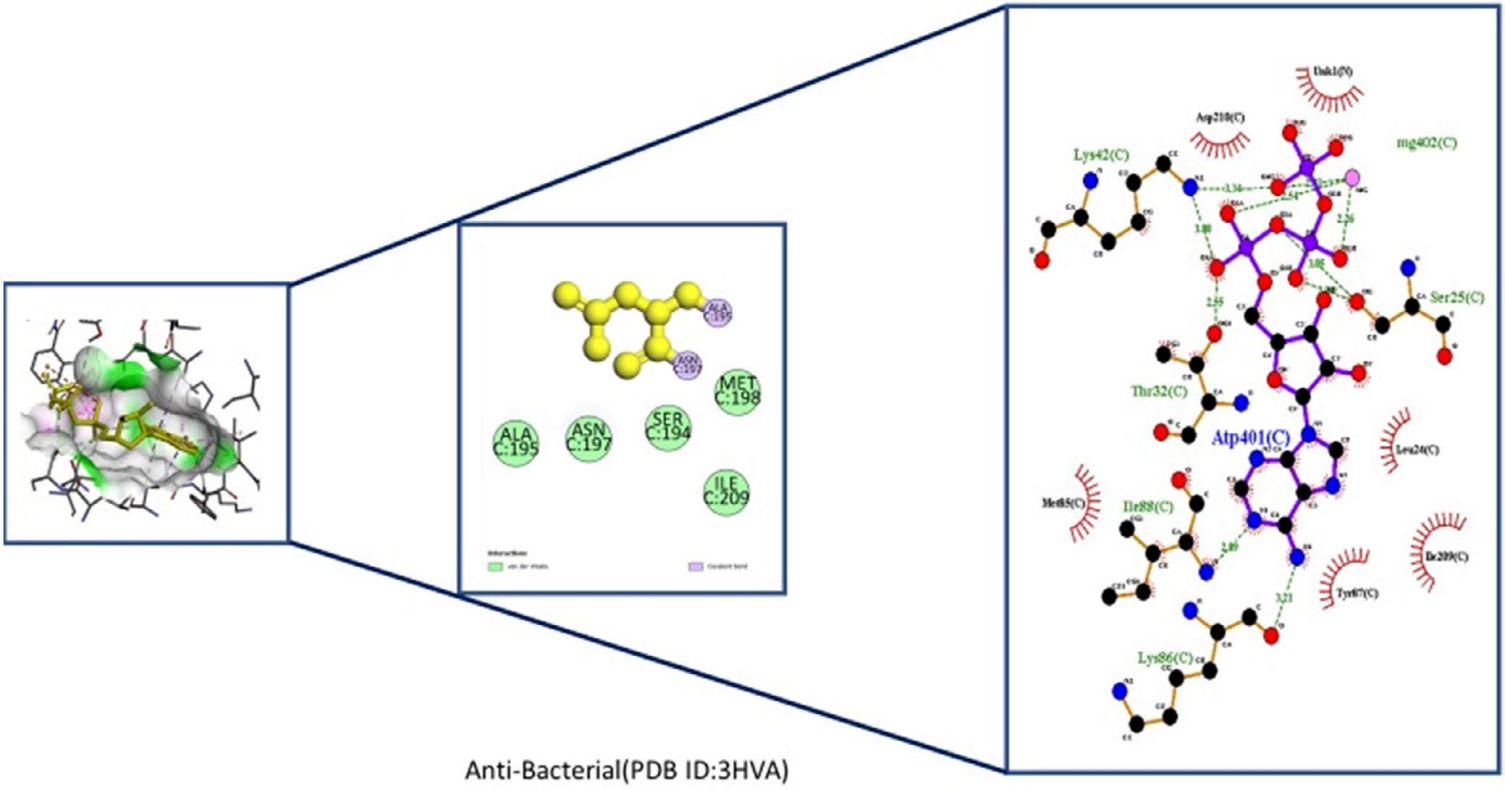
Ligplot Analysis, 3D and 2D binding types of (1aS,4aR,8aR)‐4a,8,8‐trimethyl‐1a,3,4,5,6,7‐hexahydro‐1H‐cyclopropa[j]naphthalen‐2‐one binding with specific amino acids of anti‐bacterial protein (ID: 3HVA).

Insights into sedative, hypoglycemic, cytotoxic, antioxidant, anti‐inflammatory, and antibacterial Potential: The molecular docking studies revealed that 2‐fluoro‐N‐[[(2‐fluorobenzoyl) amino]‐(3methoxyphenyl)methyl]benzamide exhibited high binding affinities against both the sedative (PDB ID: 6X3X) (Table 7, Figure 10) and hypoglycemic (PDB ID: 5YW7) (Table 7, Figure 11) target proteins, with docking energies of −6.1 and −7.4 kcal/mol, respectively, being better than the standard drugs Diazepam (−5.9 kcal/mol) and Glibenclamide (−5.9 kcal/mol). For cytotoxicity activity (PDB ID: 1NMS), Tetradecanoic acid, 10,13‐dimethyl‐, methyl ester had a binding energy of −5.5 kcal/mol (Table 7, Figure 12), comparable to that of Etoposide (−5.3 kcal/mol). For antioxidant activity (PDB ID: 1DNU), 3‐[(Z)‐heptadec‐10‐enyl]phenol had a docking score of −6.2 kcal/mol (Table 7, Figure 13), which is slightly higher than that of the control compound Quercetin (−6.1 kcal/mol). Additionally, 8‐(2‐octylcyclopropyl) octanal exhibited a binding activity of −7.3 kcal/mol toward the anti‐inflammatory target (PDB ID: 6Y3C) (Table 7, Figure 14), which is greater than that of Diclofenac (−6.2 kcal/mol). Finally, the antibacterial molecule (1aS,4aR,8aR)‐4a,8,8‐trimethyl‐1a,3,4,5,6,7‐hexahydro‐1H‐cyclopropa[j]naphthalen‐2‐one was found to have a docking value of −6.6 kcal/mol (Table 7, Figure 15), corresponding to PDB ID: 3HVA, which was slightly higher than that of Penicillin (−6.3 kcal/mol). Collectively, these findings suggest that the selected phytoconstituents possess binding affinities comparable to or greater than those of the reference drugs and are suitable as potential new lead molecules for future experimental verification.

**TABLE 7:** Molecular docking scores of ligand‐target interactions in six protein complexes.

Activity (PDB)	Ligand name	Binding affinity (kcal/mol)	Bond name	Amino acids residues
Sedative (6X3X)	2‐fluoro‐N‐[[(2‐fluorobenzoyl) amino]‐(3‐methoxyphenyl)methyl]benzamide (ID‐3291283)	−6.1	Van der Waals	ARG K:50, TYR K:35, GLU K:46
			Conventional hydrogen bond	TYP K:47
			Alkyl	LEU K:45, VAL K:37, TRP K:108
			Halogen (Florine)	ASP K:61
Hypoglycemic (5YW7)	2‐fluoro‐N‐[[(2‐fluorobenzoyl) amino]‐(3‐methoxyphenyl)methyl]benzamide (ID‐3291283)	−7.4	Van der Waals	SER B:1238, LEU B:1242, ASN B:1245, ARG B:1300
			Conventional hydrogen bond	ARG B:1245
			Halogen	ASN B:437
Cytotoxic (1NMS)	Tetradecanoic acid, 10,13‐dimethyl‐, methyl ester (ID:554145)	−5.5	Van der Waals	SER A:209, SER A:249, PHE A:256, ALA A:162, SER A:120
			Conventional hydrogen bond	PHE A:250, GLY A:122, CYS A:163, TRP A:214, ASN A:208
			Pi–Pi stacked	TYP A:214
			Covalent bond	CYS A:163
Antioxidant (1DNU)	3‐[(Z)‐heptadec‐10‐enyl] phenol (ID‐44575468)	−6.2	Van der Waals	VAL A:30, ALA A:35, LEU A:33
			Conventional hydrogen bond	TRP A:32
Anti‐inflammatory (6Y3C)	8‐(2‐octylcyclopropyl) octanal (ID‐550143)	−7.3	Van der Waals	ASN A:149, ILE A:194, LYS A:213, ASP A:290
			Conventional hydrogen bond	LYS A:196
			Covalent bond	ASN A:149
Antibacterial (3HVA)	(1aS,4aR,8aR)‐4a,8,8‐trimethyl‐1a,3,4,5,6,7‐hexahydro‐1H‐cyclopropa[j]naphthalen‐2‐one (ID‐3291283)	−6.6	Van der Waals	ALA C:195, ASN C:197, SER C A:197
			Covalent bond	ASN C:197, ALA C:195

#### 3.10.2 ADME Analysis

The pharmacokinetic properties and drug‐likeness of the phytochemicals were evaluated using SwissADME, and the results were summarized in Table 8.

**TABLE 8:** ADME analysis of *Lannea coromandelica* compounds.

Compound name	Lipinski Rules				Lipinski's violation ≤ 1	Veber's rules		
	MW (g/mol) < 500	HBA < 10	HBD < 5	Log *p* ≤ 5		n RB ≤ 10	TPSA ≤ 140 (Å^2^)	BBB
Cyclopropaneoctanal, 2‐octyl—	280	1	0	4.56	1	15	17.07	0.55
Z,Z‐6,28‐Heptatriactontadien‐2—	530	1	0	—	—	32	17.07	—
Z,Z‐6,27‐Hexatriactontadien‐2—	516	1	0	—	—	31	17.07	—
Methyl 11‐hexadecenoate	268	2	0	4.27	1	14	26.30	0.55
Tetradecanoic acid, 10,13‐dimethyl‐, methyl ester	270	2	0	4.64	1	13	26.30	0.55
n‐Hexadecanoic acid (Palmitic acid)	256	2	1	3.85	1	14	37.30	0.85
n‐Propyl 9,12‐octadecadienoate	322	2	0	—	—	17	26.30	—
13‐Octadecenoic acid, methyl ester	296	2	0	4.77	1	16	26.30	0.55
n‐Propyl 11‐octadecenoate	324	2	0	5.24	1	18	26.30	0.55
Dodecyl isothiocyanate	227	1	0	4.17	1	11	44.45	0.55
(Z)‐3‐(Heptadec‐10‐en‐1‐yl)phenol	330	1	1	4.97	1	15	20.23	0.55
Trans‐13‐docosenamide (Erucamide)	337	1	1	5.10	1	19	43.09	0.55
p‐Toluenesulfonamide, 2TBDMS derivative	399	3	0	3.88	0	6	45.76	0.55
2,4‐ditert‐butylphenol	206	—	—	—	—	—	—	—
Benzamide, 2‐fluoro‐N‐(2‐fluorobenzoyl)—	415	4	0	3.24	1	5	37.38	0.55
Acetic acid, 11‐iodomethyl—	594	2	0	5.31	2	3	26.30	0.17
γ‐Sitosterol	414	1	1	5.07	1	6	20.23	0.55
Hexadecanoic acid, ethyl ester	284	2	0	4.65	1	16	26.30	0.55

#### 3.10.3 Toxicity Evaluation

The toxicity profiles of the docked phytochemicals were evaluated by analyzing multiple parameters, including immunotoxicity, toxicity class, LD_50_, hepatotoxicity, cytotoxicity, carcinogenicity, and mutagenicity. The results of this comprehensive in silico toxicological assessment are presented in Table 9, providing critical insights into the safety and potential risk factors associated with these compounds.

**TABLE 9:** Toxicity evaluation of *Lannea coromandelica* compounds.

Compound name	Predicted LD_50_ (mg/kg)	Predicted toxicity class	Hepatotoxicity	Carcinogenicity	Mutagenicity	Immunotoxicity	Cytotoxicity
Cyclopropaneoctanal, 2‐octyl—	5000	5	−	−	−	−	−
Z,Z‐6,28‐Heptatriactontadien‐2—	15,000	6	−	−	−	−	−
Z,Z‐6,27‐Hexatriactontadien‐2—	15,000	6	−	−	−	−	−
Methyl 11‐hexadecenoate	3000	5	−	−	−	−	−
Tetradecanoic acid, 10,13‐dimethyl‐, methyl ester	5000	5	−	−	−	−	−
n‐Hexadecanoic acid (Palmitic acid)	900	4	−	−	−	−	−
n‐Propyl 9,12‐octadecadienoate	2000	6	−	+	−	−	−
13‐Octadecenoic acid, methyl ester	3000	5	−	−	−	−	−
n‐Propyl 11‐octadecenoate	339	4	−	+	−	−	−
Dodecyl isothiocyanate	1600	4	−	−	−	−	−
(Z)‐3‐(Heptadec‐10‐en‐1‐yl)phenol	2830	5	−	−	−	+	−
Trans‐13‐docosenamide (Erucamide)	750	4	−	−	−	−	−
p‐Toluenesulfonamide, 2TBDMS derivative	283	3	−	−	−	−	−
2,4‐ditert‐butylphenol	700	4	−	−	−	−	−
Benzamide, 2‐fluoro‐N‐(2‐fluorobenzoyl)—	1500	4	−	+	−	−	−
Acetic acid, 11‐iodomethyl—	13,430	6	−	+	−	+	−
γ‐Sitosterol	890	4	−	−	−	+	−
Hexadecanoic acid, ethyl ester	5000	5	−	+	−	−	−

## 4 Discussion

Despite advances in modern medicine, many drugs remain costly, unsafe, or cause side effects (Niklas Norén and Edwards 2009). To address this, researchers are documenting traditional herbal knowledge for future healthcare use (Süntar 2020). However, scientific validation is still limited, and further studies are needed to confirm the safety and efficacy of these remedies (Ur Rehman et al. 2021). To address this, the current study was conducted to identify the various bioactive components of the ME and to unveil its multi‐target potential through a series of in vitro and in vivo assays. The investigation ultimately uncovers its potential as an antioxidant, cytotoxic, antidiabetic, antibacterial, anti‐inflammatory, and sedative agent.

Qualitative phytochemical screening revealed that ME contains many phytochemicals, including alkaloids, flavonoids, saponins, tannins, phenolic compounds, glycosides, carbohydrates, proteins, amino acids, acidic compounds, phytosterols, steroids, and terpenes. Those phytoconstituents are therapeutically active and show efficacy against several disease conditions (Bose et al. 2020).

GC–MS analysis of ME identified 20 phytochemicals, including fatty acids and their esters (e.g., methyl 11‐hexadecenoate, hexadecanoic acid [palmitic acid], and hexadecanoic acid ethyl ester), long‐chain ketones/aldehydes (e.g., cyclopropaneoctanal and 2‐octyl‐), phenolic derivatives ((Z)‐3‐(heptadec‐10‐en‐1‐yl)), sterols (γ‐sitosterol), amide derivatives (trans‐13‐docosenamide/erucamide), and a few other terpenoid/alkyl derivatives. This chemical diversity suggests multiple bioactive properties, supporting further exploration for therapeutic and nutritional applications. This array of bioactive constituents was identified by GC–MS to include fatty acids and esters (palmitic acid, methyl/propyl octadecenoates), which have previously been characterized to possess antioxidant and anti‐inflammatory activity, phenolic compounds (2,4‐di‐tert‐butylphenol; (Z)‐3‐(heptadec‐10‐en‐1‐yl)phenol) implicated in antimicrobial and cytoprotective activity (Rouvier et al. 2024), and phytosterols (γ‐sitosterol) that are believed to exert anti‐inflammatory and metabolic regulatory effects (Vezza et al. 2020). Interestingly, dodecyl isothiocyanate, and long‐chain unsaturated amides (e.g., erucamide) are relatively less studied scaffolds that could provide lead compounds for future drug discovery efforts directed toward elucidating mechanisms and identifying targets.

The quantitative analysis disclosed that ME contains 35 ± 1.03 mg/g of total phenolics and 11.57 ± 0.37 mg/g of flavonoids. Phenolic compounds exert a range of pharmacological effects, including antioxidant and antibacterial activities. Moreover, extensive research has shown that phenolics and flavonoids are essential for the immune system, as they neutralize free radicals, offer cardiovascular protection, diminish inflammation, and have anti‐cancer properties (Mutha et al. 2021). Phenolic compounds have been recognized for their diverse pharmacological functions, especially for their remarkable antioxidant and antibacterial properties (Sun and Shahrajabian 2023).

ME showed strong, dose‐dependent antioxidant activity in DPPH and ABTS assays, with maximum scavenging of 87.45% (DPPH, IC_50_ 48.94 μg/mL) and 85.43% (ABTS, IC_50_ 31.49 μg/mL), indicating its potential to counter oxidative stress linked to various health conditions, including cancer, diabetes, cardiovascular and inflammatory conditions, and aging (Reddy 2023). Antioxidants can alleviate oxidative stress by neutralizing free radicals and prohibiting lipid peroxidation, thereby demonstrating their potential efficacy against these ailments (Firuzi et al. 2011). Molecular docking revealed that 3‐[(Z)‐heptadec‐10‐enyl] phenol exhibited a slightly higher binding affinity (−6.2 kcal/mol) for the target protein (PDB: 1DNU) compared to quercetin (−6.1 kcal/mol), suggesting its potential as a potent antioxidant agent. The compound formed stable hydrophobic interactions with four key amino acid residues in the active site—VAL A:30, ALA A:35, LEU A:33, and TRP A:32—which are critical for ligand stabilization and functional modulation. These interactions support the observed in vitro antioxidant activity and provide a plausible structural basis for the compound's ability to mitigate oxidative stress by potentially interfering with redox‐sensitive pathways or free radical generation.

ME exhibited significant α‐amylase inhibition (IC_50_ 93.09 μg/mL) compared to acarbose (IC_50_ 44.10 μg/mL), likely due to active phytochemicals like flavonoids and phenols contributing to its hypoglycemic effect (Marella 2017). Given that amylase is a crucial enzyme for carbohydrate digestion and plays a role in regulating and metabolizing glucose after a meal, it is a suitable target for treating insulin resistance (Agarwal and Gupta 2016). One therapeutic strategy for managing postprandial (PP) hyperglycemia in diabetes mellitus involves delaying carbohydrate digestion and absorption by inhibiting key enzymes such as α‐amylase. Inhibition of α‐amylase reduces the rapid breakdown of starch into absorbable sugars, thereby blunting post‐meal glucose spikes (Conforti et al. 2005). Clinically, synthetic inhibitors like acarbose, a complex oligosaccharide that targets pancreatic α‐amylase, are used for this purpose; however, they often cause gastrointestinal side effects, including abdominal discomfort, diarrhea, and flatulence, due to undigested carbohydrates fermenting in the colon (Narkhede et al. 2011). Our findings demonstrate that *L. coromandelica* exhibits significant in vitro α‐amylase inhibitory activity, suggesting its potential as a natural alternative. Although the precise mechanism of plant‐derived α‐amylase inhibition remains incompletely understood, it is hypothesized that bioactive constituents—particularly flavanols—may interact with the enzyme's active site or induce conformational changes that impair its function (Kim et al. 2000). *In silico* docking confirmed ME's antidiabetic potential, with 2‐fluoro‐N‐[[(2‐fluorobenzoyl)amino]‐(3‐methoxyphenyl)methyl]benzamide binding pancreatic α‐amylase at −7.4 kcal/mol, stronger than glibenclamide (−5.9 kcal/mol). Further studies are needed to elucidate the exact molecular interactions and to evaluate efficacy and safety in vivo.

Brine shrimp and Trypan blue assays showed ME's cytotoxicity, with 93.33% mortality at 100 μg/mL and LC_50_ of 36.22 μg/mL (colchicine LC_50_ 27.57 μg/mL), likely due to flavonoids and phenolic compounds with anticancer potential (Batra and Sharma 2013). Trypan blue assay showed ME reduced HeLa cell viability to < 5%, compared to 100% in the negative control and > 95% in the positive control, indicating its potential to induce apoptosis or inhibit cancer cell proliferation (Khan and Uddin 2021). Brine shrimp lethality and HeLa cell assays provide useful preliminary insights into cytotoxicity, but they differ in specificity compared to the MTT assay. The brine shrimp assay is rapid and inexpensive, offering a general indication of toxicity, though lacking direct human relevance (Waghulde et al. 2019). HeLa cell assays, by contrast, employ human cancer cells and thus yield more biologically meaningful data, though results depend on the chosen endpoint (Landry et al. 2013). The MTT assay remains the established standard, delivering sensitive, quantitative, and reproducible measures of cell viability (Grela et al. 2018). Together, brine shrimp and HeLa assays serve as effective screening tools, while MTT provides a reliable benchmark for validating cytotoxicity findings. Future work will include MTT or similar assays to validate and refine the cytotoxic profile suggested by the BSLA and HeLa cell line results.

ME showed moderate antibacterial activity at 50 μg/mL, with inhibition zones of 10, 12, 11, and 14 mm against *S. pyogenes* , *S. aureus* , *P. aeruginosa* , and *P. vulgaris* , respectively, compared to Pefloxacin (5 μg/mL: 21, 22, 19, and 20 mm, respectively), while no activity was observed against *E. coli* . Bioactive ingredients such as flavonoids and phenolics, which are known for their therapeutic characteristics, are responsible for the antibacterial actions (Tyagi et al. 2015) (Shamsudin et al. 2022). Multiple investigations have highlighted the importance of these chemicals in improving a variety of biological activities, including antibacterial, antiviral, and anti‐inflammatory properties (Sangeetha et al. 2016; Rahman et al. 2022; Rauha et al. 2000). The lack of antibacterial activity of the extract against *E. coli* can be attributed to the structural and physiological characteristics of Gram‐negative bacteria, particularly their complex outer membrane. *E. coli* possesses a double‐membrane envelope: an inner cytoplasmic membrane and an outer membrane rich in lipopolysaccharides (LPS), which acts as a formidable permeability barrier. This outer membrane restricts the penetration of many hydrophobic or high‐molecular‐weight compounds, such as certain phenolics, flavonoids, or tannins commonly found in plant extracts, preventing them from reaching intracellular targets (Nikaido 2003; Okere et al. 2018). Docking studies showed (1aS,4aR,8aR)‐4a,8,8‐trimethyl‐1a,3,4,5,6,7‐hexahydro‐1H‐cyclopropa[j]naphthalen‐2‐one bound Gyrase A (3HVA) at −6.6 kcal/mol, stronger than penicillin (−6.3 kcal/mol), suggesting ME compounds could inhibit bacterial DNA replication and serve as potential antibacterial agents.

ME demonstrated significant in vivo anti‐inflammatory activity, as evidenced by reduced carrageenan‐induced paw edema. Inflammation is the body's innate response to harmful stimuli. By inhibiting the enzymatic conversion of arachidonic acid into a wide range of pro‐inflammatory prostaglandins (e.g., PGE_2_), COX‐2 inhibition reduces vascular permeability (Ricciotti and Fitzgerald 2011), leukocyte infiltration, and the sensitization of nociceptors responsible for the edema, pain, and fever (Islam et al. 2025). The likely mechanisms underlying the observed anti‐inflammatory effects involve either direct competitive binding of active constituents to the COX‐2 catalytic site and/or suppression of COX‐2 expression via NF‐κB/MAPK pathway modulation, ultimately resulting in decreased prostaglandin biosynthesis (Desai et al. 2018).

The infusion of carrageenan into a mouse's paw initiates the synthesis of prostaglandins, leading to the development of inflammation via an indirect mechanism (Lopes et al. 2020). Nonsteroidal anti‐inflammatory drugs (NSAIDs) function by reducing the synthesis of prostaglandins, which are substances associated with inflammation and edema. This is achieved through the inhibition of an enzyme known as COX‐2. ME (200 and 400 mg/kg) significantly reduced paw edema, with 4‐h inhibition rates of 32.08% and 33.97%, respectively, surpassing diclofenac (29.72%, *p* < 0.001), suggesting potential COX‐mediated anti‐inflammatory effects (Vane and Botting 1996). In silico studies showed 8‐(2‐octylcyclopropyl)octanal had higher binding energy (−7.3 kcal/mol) to the anti‐inflammatory target (PDB: 6Y3C) than diclofenac (−6.2 kcal/mol), suggesting it may be a more effective plant‐derived alternative for inflammation inhibition (Aswad et al. 2018).

The open field assay showed that ME (200 and 400 mg/kg) significantly reduced locomotor activity, indicating a potent sedative effect. At 120 min, ME 200 showed 0 ± 0 mobility, while ME 400 and diazepam showed 11.2 ± 5.32 and 12 ± 1 (*p* < 0.001), demonstrating its effectiveness as a CNS depressant comparable to diazepam (Mandelli et al. 1978). Hole Cross tests showed ME's sedative effect, with minimal movement at 120 min: 0.4 ± 0.24 (ME 400), 1 ± 0.55 (ME 200), and 1 ± 0.32 (diazepam, *p* < 0.001), indicating dose‐independent sedation likely via GABAergic modulation (Szabadi 2006). These neuropharmacological effects support ME as a CNS drug candidate. In silico docking showed the bioligand 2‐fluoro‐N‐[[(2‐fluorobenzoyl)amino]‐(3‐methoxyphenyl)methyl]benzamide had higher binding energy (−6.1 kcal/mol) to PDB 6X3X than diazepam (−5.9 kcal/mol), suggesting stronger GABA‐inhibitory potential (Mandelli et al. 1978). ME may have sedative‐hypnotic potential as seen in the open‐field, since the reduction of locomotor activity with doses of ME comparable to diazepam appears to be dose‐dependent; however, the significant immobility that was observed could also be more indicative of non‐specific motor suppression or CNS toxicity as opposed to physiological sedation. Hence, mechanistic validation (e.g., GABAergic involvement), motor coordination assays (rotarod), and acute/subchronic toxicity with dose–response profiling are needed to delineate the therapeutic window and safety margin. This suggests plant derivatives could be promising alternatives for developing CNS‐targeted drugs with fewer side effects (Edewor‐Kuponiyi 2013). Overall, these proteins were selected as validated, disease‐relevant molecular targets covering key pathways of sedation, glucose regulation, apoptosis, oxidative stress, inflammation, and bacterial biofilm formation, thereby enabling a comprehensive multi‐target evaluation of the bioactive compounds through docking‐based mechanistic insight.

Collectively, ME shows strong antidiabetic, anti‐inflammatory, and sedative effects, with notable cytotoxicity and moderate antioxidant and antibacterial activity. These findings validate the ethnomedicinal use of *L. coromandelica* and highlight its potential as a source of bioactive compounds, warranting further fractionation and chemical isolation to fully explore its therapeutic prospects.

## 5 Limitations

Despite yielding important preliminary results, the current study contains some limitations. Firstly, the pharmacological assays were carried out using a crude methanol extract rather than purified bioactive compounds, which could produce either synergistic or antagonistic interactions and were not evaluated for individual interactions. Secondly, the antibacterial assays were limited to agar well diffusion assays, without calculating MIC or MBC values for more quantitative analysis. Thirdly, whereas molecular docking analysis was used to complement laboratory data, molecular dynamics simulations or enzymatic assays for targets of interest were not evaluated to support interaction predictions. Fourthly, the in vivo experiments were limited by a small number of dose levels without a detailed analysis of the dose–response relationship. Fifthly, While ME demonstrated a more pronounced reduction in locomotor activity relative to diazepam, the lack of motor coordination assessments, toxicity evaluations, and video‐based behavioral tracking precludes a conclusive distinction between sedation and genuine anxiolytic action. Subsequent investigations integrating targeted locomotor controls and comprehensive central nervous system safety evaluations would be valuable to clarify these effects. Furthermore, the identity of compounds determined by GC–MS analysis relied solely on machine matching without verification against authentic standards, and it is difficult to completely rule out the presence of impurities. These factors indicate that the study data should be treated with caution and that additional experimental investigations are needed to verify the therapeutic properties of *L. coromandelica*.

## 6 Conclusions

Despite its well‐documented ethnomedicinal applications, the fruit of *L. coromandelica* has remained significantly underexplored in scientific literature. This study presents a comprehensive, exploratory pharmacological evaluation of its methanolic fruit extract (ME) through an integrated in vitro, in vivo, and in silico approach. The extract demonstrated notable antioxidant, cytotoxic, antidiabetic, antibacterial, anti‐inflammatory, and sedative activities, providing scientific support for its traditional therapeutic uses. Phytochemical profiling via GC–MS revealed a range of bioactive compounds that likely underpin the observed biological effects. Furthermore, molecular docking studies against key target proteins corroborated the experimental findings by predicting strong binding affinities of major constituents. However, as this study employed a crude extract and relied on preliminary screening assays, the results should be interpreted cautiously, and active principles have not yet been isolated or fully characterized. Nevertheless, the collective evidence strongly underscores the therapeutic potential of *L. coromandelica* fruit and provides a compelling rationale for future work involving bioactivity‐guided fractionation, mechanistic studies, toxicological profiling, and preclinical validation to advance its development as a standardized phytopharmaceutical agent.

## 7 Future Recommendations

Subsequent research should prioritize the bioactivity‐directed isolation and characterization of individual constituents to pinpoint their specific active compounds and potential synergistic effects. A more rigorous antimicrobial evaluation should incorporate quantitative assays, such as minimum inhibitory and bactericidal concentrations (MIC/MBC), alongside testing against a broader panel of microbial strains. Furthermore, computational predictions ought to be substantiated through molecular dynamics modeling and targeted enzyme assays to verify molecular target interactions. Given the pronounced cytotoxicity observed against HeLa cells (< 5% viability), future investigations should prioritize dose–response profiling, IC_50_ determination, and mechanistic studies to elucidate whether cell death occurs via apoptosis or necrosis. Validation with complementary viability assays (e.g., MTT, flow cytometry) and pathway‐specific analyses will be essential to confirm these preliminary findings. Additional studies should also incorporate thorough dose–response evaluations and comprehensive central nervous system profiling, including motor function and behavioral testing to clearly differentiate between anxiolytic and sedative properties. Comprehensive safety assessments and the verification of GC–MS results against certified reference standards are equally critical. Ultimately, rigorously planned preclinical and human trials will be essential to advance *L. coromandelica* toward development as a standardized phytopharmaceutical.

## Author Contributions

**Priota Islam Meem:** conceptualization, writing – original draft, validation, formal analysis, data curation, investigation, software. **Amit Kumar Dam:** writing – review and editing, formal analysis. **Zobayed Islam:** investigation, writing – original draft, methodology, software. **Khurram Murad Mojidee:** methodology, formal analysis. **Mohammad Badrudduja:** formal analysis, methodology. **Md. Jahirul Islam Mamun:** investigation, writing – original draft, methodology, software. **Nazmul Hasan Eshaque:** methodology, investigation, writing – original draft, writing – review and editing, formal analysis. **S. M. Moazzem Hossen:** methodology, conceptualization, validation, writing – original draft, writing – review and editing, supervision, project administration.

## Funding

The authors have nothing to report.

## Conflicts of Interest

The authors declare no conflicts of interest.

## Data Availability

Data will be made available on request.
